# The Cu(II) Reductase RclA Protects *Escherichia coli* against the Combination of Hypochlorous Acid and Intracellular Copper

**DOI:** 10.1128/mBio.01905-20

**Published:** 2020-09-29

**Authors:** Rhea M. Derke, Alexander J. Barron, Caitlin E. Billiot, Ivis F. Chaple, Suzanne E. Lapi, Nichole A. Broderick, Michael J. Gray

**Affiliations:** a Department of Microbiology, School of Medicine, University of Alabama at Birmingham, Birmingham, Alabama, USA; b Department of Molecular and Cell Biology, University of Connecticut, Storrs, Connecticut, USA; c Department of Radiology, School of Medicine, University of Alabama at Birmingham, Birmingham, Alabama, USA; d Institute for Systems Genomics, University of Connecticut, Storrs, Connecticut, USA; University of Arizona

**Keywords:** copper, hypochlorous acid, oxidative stress, reactive chlorine

## Abstract

Enterobacteria, including Escherichia coli, bloom to high levels in the gut during inflammation and strongly contribute to the pathology of inflammatory bowel diseases. To survive in the inflamed gut, E. coli must tolerate high levels of antimicrobial compounds produced by the immune system, including toxic metals like copper and reactive chlorine oxidants such as hypochlorous acid (HOCl). Here, we show that extracellular copper is a potent detoxifier of HOCl and that the widely conserved bacterial HOCl resistance enzyme RclA, which catalyzes the reduction of copper(II) to copper(I), specifically protects E. coli against damage caused by the combination of HOCl and intracellular copper. E. coli lacking RclA was highly sensitive to HOCl when grown in the presence of copper and was defective in colonizing an animal host. Our results indicate that there is unexpected complexity in the interactions between antimicrobial toxins produced by innate immune cells and that bacterial copper status is a key determinant of HOCl resistance and suggest an important and previously unsuspected role for copper redox reactions during inflammation.

## Abstract (precis)

During infection and inflammation, the innate immune system uses antimicrobial compounds to control bacterial populations. These include toxic metals, like copper, and reactive oxidants, including hypochlorous acid (HOCl). We have now found that RclA, a copper(II) reductase strongly induced by HOCl in proinflammatory Escherichia coli and found in many bacteria inhabiting epithelial surfaces, is required for bacteria to resist killing by the combination of intracellular copper and HOCl and plays an important role in colonization of an animal host. This finding indicates that copper redox chemistry plays a critical and previously underappreciated role in bacterial interactions with the innate immune system.

## INTRODUCTION

Inflammatory bowel diseases (IBDs), such as Crohn’s disease and ulcerative colitis, are a growing health problem (1) and are associated with dramatic changes in the composition of the gut microbiome (2–6). Patients with IBDs have increased proportions of proteobacteria, especially Escherichia coli and other *Enterobacteriaceae*, in their gut microbiomes, which is thought to contribute to the progression of disease (7–10). The bloom of enterobacteria in the inflamed gut is driven by increased availability of respiratory terminal electron acceptors (e.g., oxygen, nitrate, and trimethylamine N-oxide) and carbon sources (e.g., ethanolamine and mucin), which E. coli and other facultative anaerobes can use to outcompete the obligate anaerobes (*Bacteroides* and *Clostridia*) that dominate a healthy gut microbiome (2, 3). In addition to these nutritional changes in the gut environment, inflammation also leads to infiltration of innate immune cells (e.g., neutrophils) into the lumen of the gut (11) and an associated increased production of antimicrobial compounds by the innate immune system (9, 10, 12), which also impact the bacterial community in the gut. These include antimicrobial peptides, toxic metals (e.g., copper), reactive oxygen species (ROS), reactive nitrogen species, and reactive chlorine species (RCS) (3, 13, 14). Since bacteria living in an inflamed gut are likely to be exposed to substantially increased levels of these toxins, the differential survival of proteobacteria during long-term inflammation suggests that E. coli may have evolved better mechanisms to resist these stresses than other types of commensal bacteria.

Macrophages use copper as an antimicrobial agent and copper levels rise in inflamed tissues, although the exact mechanism(s) by which copper kills bacteria are not yet fully understood (15–17). RCS are highly reactive oxidants produced by neutrophils and are potent antibacterial compounds (18, 19) that have been reported to play a role in controlling intestinal bacterial populations (20–22). E. coli does not efficiently survive being phagocytosed by neutrophils (23, 24), but the release of RCS-generating myeloperoxidase from neutrophils in the inflamed gut (11) means that E. coli is exposed to increased levels of RCS in that environment. The RCS response of E. coli is complex and incompletely understood (19, 25–28) but characteristically involves repair of damaged cellular components, often proteins (19). Protein-stabilizing chaperones are upregulated during RCS stress, including Hsp33 (29, 30) and inorganic polyphosphate (28, 31, 32), and enzymes are expressed that repair oxidized proteins, including periplasmic methionine sulfoxide reductase (MsrPQ) (33) and the chaperedoxin CnoX (34). E. coli has multiple HOCl-sensing regulators, including YedVW (which regulates MsrPQ) (33), NemR (a regulator of NemA and GloA, which detoxify reactive aldehydes) (25), HypT (which regulates cysteine, methionine, and iron metabolism) (27), and RclR, the RCS-specific activator of the *rclABC* operon (26).

**FIG 1:**
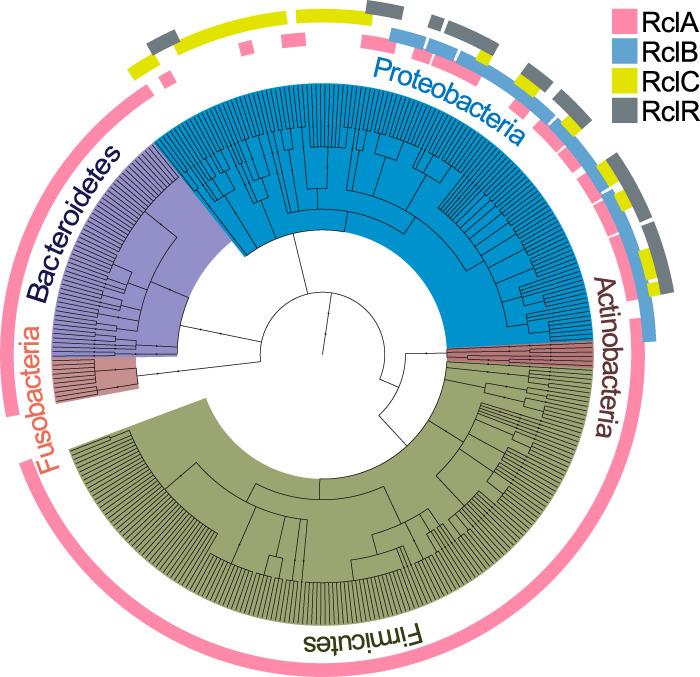
RclA is widely conserved among bacteria that colonize epithelial surfaces. A phylogenetic tree constructed from amino acid sequence alignments of RclA (pink, 284 species), RclB (blue, 61 species), RclC, (yellow, 49 species), and RclR (gray, 43 species), relative to the respective proteins in E. coli MG1655. Phyla are indicated by color: *Actinobacteria* (brown), *Bacteroidetes* (purple), *Firmicutes* (green), *Fusobacteria* (mauve), and *Proteobacteria* (blue). See Data Set S1 in the supplemental material for lists of each hit used in the phylogenetic tree. A tree graphic was made using the interactive tree of life (108).

The *rclA* gene of E. coli encodes a predicted cytoplasmic flavin-dependent oxidoreductase, is upregulated >100-fold in the presence of RCS (26, 35), and protects against killing by HOCl via a previously unknown mechanism (26). RclA is the most phylogenetically conserved protein of the Rcl system (Fig. 1) and is found almost exclusively in bacteria known to colonize epithelial surfaces (see Data Set S1 in the supplemental material), suggesting that it may play an important role in host-microbe interactions in many species. Bacteria encoding RclA homologs include Gram-negative species (e.g., Salmonella enterica), Gram-positive species (e.g., Streptococcus sanguinis), obligate anaerobes (e.g., Clostridium perfringens), facultative anaerobes (e.g., Staphylococcus aureus), pathogens (e.g., Enterococcus faecalis), commensals (e.g., Bacteroides thetaiotaomicron), and probiotics (e.g., Lactobacillus reuteri) (36), suggesting that RclA’s function may be broadly conserved and not specific to a single niche or type of host-microbe interaction. RclR, RclB, and RclC are much less widely conserved and are found only in certain species of proteobacteria, primarily members of the *Enterobacteriaceae* (Fig. 1; see Data Set S1 in the supplemental material). RclB and RclC both contribute to HOCl resistance, but their mechanistic roles have yet to be determined (26). RclB is predicted to be located in the periplasm, and RclC is predicted to be an integral inner membrane protein.

10.1128/mBio.01905-20.1 DATA SET S1 Bacterial species containing homologs of the Rcl proteins of E. coli. Homologs of the RclA, RclB, RclC, and RclR proteins of E. coli included in Fig. 1, as identified by BLAST: RclA (BLAST E-value < 1 × 10^−90^ in 284 species), RclB (BLAST E-value < 1 × 10^−1^ in 61 species), RclC, (BLAST E-value < 1 × 10^−80^ in 49 species), and RclR (BLAST E-value < 1 × 10^−40^ in 43 species). Download Data Set S1, XLSX file, 0.03 MB. Copyright © 2020 Derke et al. 2020 Derke et al. This content is distributed under the terms of the Creative Commons Attribution 4.0 International license.

Here, we have now determined that RclA is a thermostable, HOCl-resistant copper(II) reductase that is required for efficient colonization of an animal host and protects E. coli specifically against the combination of HOCl and intracellular copper, possibly by preventing the formation of highly reactive Cu(III). We also found that, surprisingly, extracellular copper effectively protects bacteria against killing by HOCl both in cell culture and in an animal colonization model. These findings reveal a previously unappreciated interaction between two key inflammatory antimicrobial compounds and a novel way in which a commensal bacterium responds to and resists the combinatorial stress caused by copper and HOCl.

(This article was submitted to an online preprint archive [37].)

## RESULTS

### RclA contributes to HOCl resistance and host colonization.

An *rclA* mutant of E. coli is more susceptible to HOCl-mediated killing than is the wild type (26). To expand on these results, we utilized here a growth curve-based method to measure sensitivity to sublethal HOCl stress by quantifying changes in the lag-phase extension (LPE) of cultures grown in the presence of HOCl. Using this method, which we have found to be considerably more reproducible than other techniques for assessing bacterial HOCl sensitivity, we observed significant increases in LPE for a Δ*rclA* mutant strain compared to the wild type grown in the presence of various concentrations of HOCl (see Fig. S1A, B, and C in the supplemental material). We observed similar trends when the assay was performed with stationary- and log-phase cells (see Fig. S1D, E, and G), so stationary-phase cultures were used for subsequent assays. Furthermore, we determined that there was no decrease in CFU after treatment with HOCl at these concentrations, indicating that LPE measures the recovery of cultures from nonlethal stress (see Fig. S1F). These results confirm previous results and further illustrate the importance of RclA in resisting HOCl-mediated oxidative stress in E. coli.

10.1128/mBio.01905-20.3 FIG S1 Growth curve method to determine lag-phase extension in the presence of HOCl. Overnight cultures of (A) MG1655 wild-type and (B) MJG0046 (Δ*rclA*) were normalized to an *A*_600_ of 0.8 in MOPS minimal media and diluted to an *A*_600_ of 0.08 in MOPS ± HOCl at the indicated concentrations. Cultures were incubated with shaking at 37°C in a Tecan Infinite M1000 plate reader with the *A*_600_ being measured every hour for 24 h (*n* = 3 to 4, ± the SD). Sensitivity was assessed by comparing lag phase extensions (LPE; difference in hours to reach *A*_600_ ≥ 0.15 from no HOCl control; vertical dotted lines) for each HOCl concentration. An example LPE calculation for wild-type grown in 148 μM HOCl shown in panel A. (C) Average LPE values for growth curves shown in panels A and B and statistics comparing wild-type and Δ*rclA* strains at all conditions. Differences in average LPE values between the strains were analyzed using two-way ANOVA with Tukey’s multiple-comparison test. HOCl growth curves were performed as in panels A and B but on log-phase cultures of each strain. To prepare the log-phase cultures, saturated overnight cultures were subcultured in MOPS glucose and grown up at 37°C with shaking at 200 rpm until *A*_600_ = 0.5. E. coli lacking *rclA* (D) took longer to reach an *A*_600_ of 0.15 relative to the wild type (E) at all the tested HOCl concentrations. Time for each sample to reach an average A_600_ of 0.15 is indicated by vertical dashed lines. The observed growth delays of the strains in the presence of HOCl did not correspond with killing, as determined by viability spot plates (C, pictures representative of three biological replicates). Viability spot plates were prepared by collecting 5-μl aliquots from each well used for growth curves 10 min after adding MOPS ± HOCl to the OD-normalized cells. Serial dilutions in PBS of the aliquots were performed, and 5 μl of the dilutions was spotted on LB plates and grown overnight at 37°C. (G) Average LPE values for growth curves shown in D and E and statistics comparing wild-type and Δ*rclA* strains at all conditions. Differences in average LPE values between the strains were analyzed using two-way ANOVA with Tukey’s multiple-comparison test. Download FIG S1, TIF file, 2.2 MB. Copyright © 2020 Derke et al. 2020 Derke et al. This content is distributed under the terms of the Creative Commons Attribution 4.0 International license.

**FIG 2:**
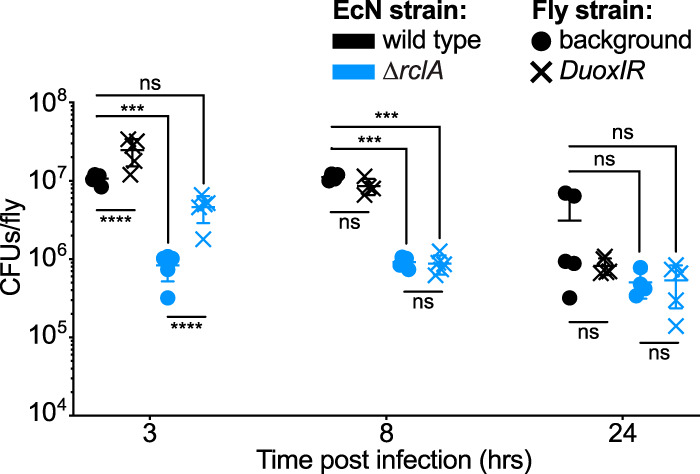
EcN lacking *rclA* colonizes D. melanogaster less effectively, and early colonization with *ΔrclA* EcN is improved in the absence of Duox-mediated oxidation. Flies were fed either wild-type or *ΔrclA* EcN (1 × 10^11^ CFU/ml), and bacterial loads were measured at the indicated times postinfection (*n* = 4 to 5, ± the SD). RCS-deficient Duox-RNAi flies were obtained from crosses of *UAS-dDuox-RNAi* with *NP1-GAL4* (enterocyte-specific driver). Statistical analysis was performed using a two-way analysis of variance (ANOVA) with Tukey’s multiple-comparison test (****, *P* < 0.0001; ***, *P* < 0.001; ns, not significant).

To directly test the role of RclA in interactions with an animal host, we examined the ability of E. coli to colonize the intestine of Drosophila melanogaster, where the presence of enterobacteria is known to stimulate antimicrobial HOCl production by the dual oxidase Duox (20, 22). Since an E. coli K-12 strain did not efficiently colonize D. melanogaster (see Fig. S2A), we used the colonization-proficient E. coli strain Nissle 1917 (EcN) (38, 39) in these experiments. The genome of EcN encodes homologs of all the known HOCl resistance genes found in E. coli K-12 (40, 41). Unlike K-12, EcN forms robust biofilms (42–45), preventing the accurate measurement of growth curves and LPE (Fig. S2B), but EcN was slightly more sensitive to killing by lethal doses of HOCl than was MG1655 (Fig. S2C and D). EcN Δ*rclA* mutants had a significant defect in their ability to colonize NP1-GAL4 D. melanogaster flies compared to wild-type EcN at 3 and 8 h postinfection (hpi) (Fig. 2, circles). This shows that *rclA* is important for EcN tolerance of host responses during early colonization.

10.1128/mBio.01905-20.4 FIG S2 Differences in fly colonization and HOCl sensitivity between E. coli K-12 and EcN. (A) EcN colonizes Canton-S D. melanogaster to a greater extent than MG1655. Flies were fed either E. coli K-12 MG1655 (K-12) or Nissle 1917 (EcN) and bacterial loads were measured (*n* = 5, ± the SD). Statistical analysis was performed using two-way ANOVA with Tukey’s multiple-comparison test (****, *P* < 0.0001; ns, not significant). (B) EcN growth curves with HOCl. EcN forms adherent growth in 96-well plates when grown with HOCl, which obstructs accurate measurement of absorbance over time. Growth curves performed as in Fig. S1A (*n* = 3 to 4, ± the SD). (C and D) EcN is more sensitive to HOCl-mediated killing than K-12. Wild-type E. coli MG1655 (K-12) (C) and Nissle 1917 (EcN) (D) were grown overnight in MOPS minimal media. A portion (500 μl) of the overnight culture was pelleted, resuspended in copper-free MOPS, and subcultured into 9.5 ml of copper-free MOPS. The cultures were then grown with shaking at 37°C to mid-log phase (*A*_600_ = 0.3 to 0.4). Once mid-log phase was reached, 1 ml of cells was aliquoted into microcentrifuge tubes for each treatment. Cells were treated by adding the indicated amounts HOCl and then incubated on a 37°C heat block for 10 min. After incubation, treatments were serially diluted in PBS, and 5-μl aliquots were spotted on LB agar plates. Plates were dried and incubated at room temperature for 2 days before imaging. Download FIG S2, TIF file, 1.3 MB. Copyright © 2020 Derke et al. 2020 Derke et al. This content is distributed under the terms of the Creative Commons Attribution 4.0 International license.

To investigate the role of host-produced RCS in the colonization defect of the *rclA* EcN mutant, we reduced the gut-specific expression of Duox in the flies using Duox-RNAi and repeated the colonization experiments. Both strains colonized significantly better at 3 hpi when Duox was knocked down in the flies (Fig. 2, “×” symbols), which was expected because *rclA* is not the only gene that contributes to HOCl resistance in E. coli (19). Importantly, the colonization defect of *ΔrclA* EcN at 3 hpi was abrogated in the *DuoxIR* flies, with CFU/fly not being significantly different from wild-type EcN colonizing flies that are able to express Duox in the gut (Fig. 2). We confirmed that HOCl production was reduced in the *DuoxIR* flies using the HOCl-sensing fluorescent probe R19-S (see Fig. S3A to C) (46). Taken together, these results show that *rclA* facilitates early colonization of an animal host and indicate that *rclA* relieves stress caused by host-produced oxidation in early stages of colonization. Duox activation and HOCl production are rapid host immune responses that occur at early stages (minutes to first few hours) of bacterial colonization of the gut (20). However, ROS and RCS production are not the only antimicrobial responses in *Drosophila*, which may explain why *rclA* is only required in early colonization. As the course of infection progresses, additional antimicrobial effectors, such as antimicrobial peptides regulated by NF-κB signaling, become more abundant (27). EcN infection induced robust production of antimicrobial peptide production in our fly model (Fig. S3D, E, and F).

10.1128/mBio.01905-20.5 FIG S3 Fly response to EcN colonization. (A) Host-produced HOCl production is silenced by Duox RNAi. Prior to treatment, flies were starved for 2 h at 29°C. (A) As an uninfected control, NP1-GAL4 female flies fed on a mixture of LB, and 100 μM R19-S was prepared in 5% sucrose. RCS production in response to EcN infection was determined by feeding a mixture of EcN (5 × 10^7^ cells) and 100 μM R19-S to NP1-GAL4 female flies (B) and NP1-GAL4-UAS-Duox^IR^ female flies (C). After 30 min of feeding on the designated mixture, all treatments were switched to a sucrose/R19-S mixture. At 90 min after the initial infection, guts were dissected in PBS and fixed in 4% formaldehyde for 50 min. Guts were mounted in Vectashield/DAPI and visualized on a Leica SP8 confocal microscope with a red emission range of 530 to 603 nm. Images presented are representative of a total of three gut tissues imaged per treatment. (D to F) The antimicrobial peptide Diptericin (Dpt) is expressed in response to oral infection with EcN in *DD;NP1* flies. Adult female flies were fed EcN (E) or a sucrose (D) control for 6 h, and gut tissues were dissected and stained with X-Gal to reveal regions of Dpt expression, method described previously (C. Neyen, A. J. Bretscher, O. Binggeli, and B. Lemaitre, Methods 68:116–128, 2014, https://doi.org/10.1016/j.ymeth.2014.02.023). (F) β-Galactosidase activity of adult female gut tissues at various time points after oral infection with EcN. The relative β-galactosidase activity is expressed as a percentage of baseline activity observed in flies fed a sterile sucrose solution. Download FIG S3, TIF file, 2.7 MB. Copyright © 2020 Derke et al. 2020 Derke et al. This content is distributed under the terms of the Creative Commons Attribution 4.0 International license.

### RclA is homologous to mercuric reductase and copper response genes are upregulated after HOCl stress in EcN.

**FIG 3:**
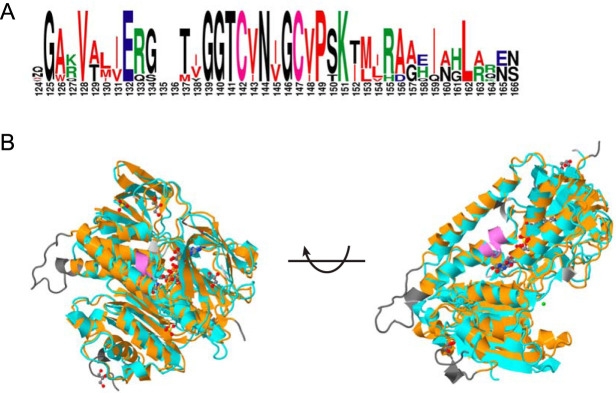
RclA and mercuric reductase (MerA) share a conserved active site and are structurally homologous. (A) Active site alignment of E. coli RclA and MerA amino acid sequences from seven bacterial species (Escherichia coli, Staphylococcus aureus, Salmonella enterica, Listeria monocytogenes, Klebsiella pneumoniae, and Serratia marcescens). Alignment was made using CLUSTAL O (1.2.4), and a graphic was made using WEBLOGO. (B) Structural alignment of MerA (orange) and RclA (cyan) with active sites in purple and nonconserved regions in gray. Polymers represented as ribbons, and ligands (FAD, SO_4_, glycerol, and Cl^–^) are represented in ball-and-stick form. Comparison and graphics made using the “sequence and structure alignment” tool (jFATCAT_rigid algorithm with input sequences 1ZK7.A and 6KGY. (B) Provided by the RCSB (109).

Although the fact that *rclA* protects E. coli from RCS was previously known (26), the mechanism by which it does so was not. Based on its homology to other flavin-dependent disulfide oxidoreductases (47), we hypothesized that RclA catalyzed the reduction of an unknown cellular component oxidized by RCS. RclA is homologous to mercuric reductase (MerA), an enzyme that reduces Hg(II) to Hg(0) (48). These sequences are particularly well conserved at the known active site of MerA, a CXXXXC motif (Fig. 3A). However, MerA has an extra N-terminal domain and two additional conserved cysteine pairs used in metal binding (48), while RclA only has one conserved cysteine pair (see Fig. S4). This indicates that if RclA does interact with metal(s), the interactions must be mediated through mechanisms different from those of MerA. While the present manuscript was in revision, Baek et al. published a crystal structure of RclA (49), and alignment of this structure with that of MerA (50) clearly illustrates the homology between these two proteins as well as the location of the MerA-specific domains (Fig. 3B).

10.1128/mBio.01905-20.6 FIG S4 Alignment of full-length RclA with MerA sequences from representative bacterial species. Alignment of E. coli RclA (ADC80840.1) and MerA amino acid sequences from seven species (Escherichia coli, ADC80840.1; Staphylococcus aureus, AKA87329.1; Salmonella enterica, ABQ57371.1; Listeria monocytogenes, PDA94520.1; Klebsiella pneumoniae, ABY75610.1; Serratia marcescens, ADM52740.1). Alignment, conservation scoring, and graphic were made using PRALINE. Download FIG S4, TIF file, 2.6 MB. Copyright © 2020 Derke et al. 2020 Derke et al. This content is distributed under the terms of the Creative Commons Attribution 4.0 International license.

**FIG 4:**
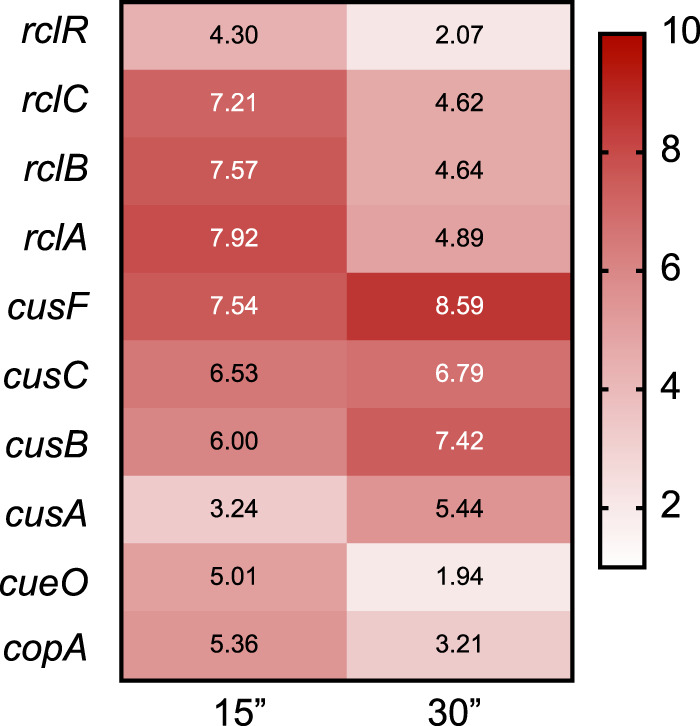
EcN upregulates copper export proteins during HOCl stress. Log_2_-fold change in EcN gene expression at 15 and 30 min after nonlethal HOCl treatment (0.4 mM), relative to a control sample taken directly before treatment. mRNA counts were measured using RNA sequencing and analyzed with Bowtie2.

HOCl-stressed E. coli K-12 downregulates genes encoding iron import systems (e.g., *fepABCD* and *fhuACDF*) and upregulates genes for zinc and copper resistance (e.g., *copA*, *cueO*, *cusC*, *zntA*, and *zupT*) (25), also suggesting metals may play some role in RCS resistance. The genome of EcN encodes the same complement of known copper resistance genes as is found in E. coli K-12 (40, 41). Transcriptomic profiling of EcN after treatment with a sublethal dose of HOCl confirmed the regulation of metal stress response genes by HOCl, including the upregulation of several genes encoding proteins involved in response to copper toxicity (Fig. 4; see also Data Set S2 in the supplemental material), despite the very small amounts of copper present in the media used in that experiment (9 nM) (51). These included members of the Cus and Cue export systems, which are factors appreciated for their role in preventing copper toxicity and importance for E. coli colonization within mammalian hosts (52–55). The expression of the *rcl* operon is not regulated by any of the known Cu-sensing transcription factors of E. coli (56) and is not affected by changes in media copper concentrations (57, 58). Our results suggested that copper might play an important role during HOCl stress for EcN. The homology between RclA and MerA and the indication that copper and HOCl responses may be connected in E. coli led us to investigate the role of *rclA* in resisting HOCl stress under growth conditions containing different amounts of copper.

10.1128/mBio.01905-20.2 DATA SET S2 Results of RNA sequencing of HOCl-treated EcN. RPKM and log_2_-fold change values for all genes in E. coli Nissle before and 15 and 30 min after treatment with 0.4 mM HOCl. Raw data are deposited under GEO series accession number GSE144068. Download Data Set S2, XLSX file, 1.4 MB. Copyright © 2020 Derke et al. 2020 Derke et al. This content is distributed under the terms of the Creative Commons Attribution 4.0 International license.

### Extracellular CuCl_2_ protects both wild-type and *ΔrclA E. coli* strains against HOCl.

**FIG 5:**
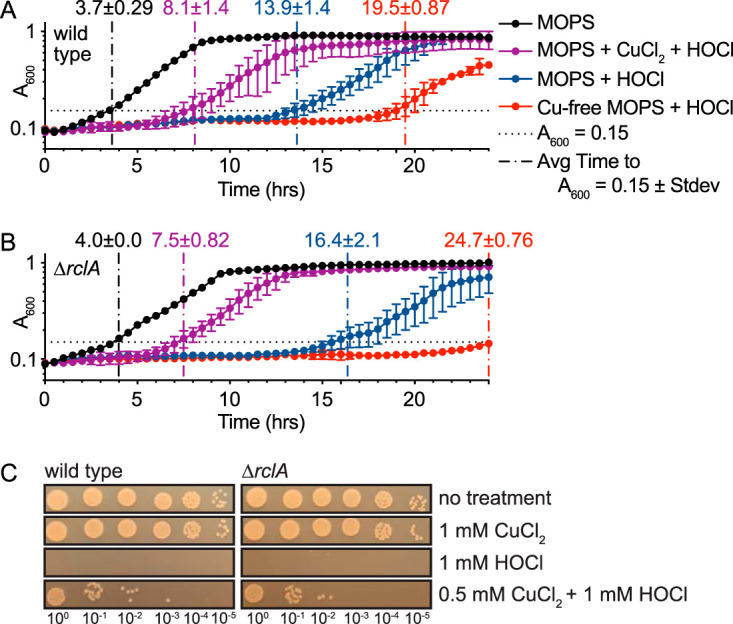
Extracellular CuCl_2_ protects both wild-type and Δ*rclA* E. coli strains against HOCl. Growth curves of E. coli K-12 wild-type (A) and Δ*rclA* (B) strains in MOPS with the addition of various combinations of HOCl (132 μM) and CuCl_2_ (10 μM) (*n* = 3, ± the SD for each treatment). Time (means ± the SD) for each sample to reach an *A*_600_ of 0.15 is indicated by vertical dashed lines. Only one of several HOCl concentrations tested is shown for simplicity (see Fig. S5A to C in the supplemental material for lag-phase extension calculations, a summary of all the conditions tested using the growth curve method, and statistics comparing LPE values shown in this figure). (C) Exogenous copper protects both wild-type and Δ*rclA* E. coli strains from lethal HOCl stress (10 min at 1 mM). Cells were treated as described in Fig. S2C and D.

How the presence of copper influences bacterial sensitivity to RCS has not been investigated before this study. However, it is important to note that Cu chemically catalyzes the decomposition of HOCl to nontoxic O_2_ and Cl^–^ (59–62). We first used growth curves in the presence of copper and HOCl to identify how combinations of HOCl and extracellular copper influenced the sensitivity of wild-type and Δ*rclA* mutant E. coli (Fig. 5A and B). As noted above, the Δ*rclA* mutant was more sensitive to HOCl in MOPS, but the sensitivity to HOCl of both strains was greatly increased when Cu was removed from the media, indicating that the low concentration of Cu present in morpholinepropanesulfonic acid (MOPS) medium (9 nM) (51) was enough to react with the added HOCl and change the sensitivity of our strains. Consistent with this, addition of 10 μM CuCl_2_ to HOCl-containing media greatly decreased sensitivity of both the wild type and the *rclA*-null mutant to sublethal HOCl stress (Fig. 5A and B; see also Fig. S5A, B, and C in the supplemental material). That copper is uniformly protective for both strains makes it likely that extracellular copper had reacted with and detoxified the HOCl before cells were inoculated into the media. To confirm this result, we tested whether the addition of exogenous copper (0.5 mM CuCl_2_) could protect E. coli against killing by a very high concentration of HOCl (1 mM). We found that treatment with 1 mM HOCl resulted in complete killing of both strains (Fig. 5C). Both strains survived several orders of magnitude better when 0.5 mM CuCl_2_ was added to the media immediately before HOCl stress (Fig. 5C). In addition, colonization of flies by EcN was enhanced when their diet was supplemented with copper (see Fig. S5D), showing that copper can influence the microbiome *in vivo*, and consistent with the model that copper detoxifies HOCl in the gut. Taken together, these results illustrate that the presence of exogenous copper strongly protects E. coli against HOCl.

10.1128/mBio.01905-20.7 FIG S5 Extracellular CuCl_2_ reduces LPE for both wild-type and Δ*rclA* E. coli grown in the presence of HOCl. (A) Average LPE values for growth curves shown in Fig. 5 and statistics comparing wild-type and Δ*rclA* at all conditions. Differences in average LPE values between the strains were analyzed using two-way ANOVA with Tukey’s multiple-comparison test. LPE determination for wild-type (B) and Δ*rclA* (C) strains grown in the presence of the indicated HOCl and extracellular CuCl_2_ conditions. Growth curves were performed as described in Fig. S1 but with supplementing the MOPS glucose with the indicated CuCl_2_ concentrations. HOCl and CuCl_2_ were mixed in MOPS minimal media prior to diluting cells *A*_600_-normalized to 0.08 into each media type. LPEs were calculated by determining the difference in time (h) to reach *A*_600_ > 0.15 for each condition relative to no stress controls. Sensitivity was assessed within strains by comparing LPEs for each HOCl/CuCl_2_ concentration (*n* = 4, ± the SD) at each HOCl concentration (two-way ANOVA for each strain with Dunnett’s multiple comparison using the no CuCl_2_ control for each HOCl concentration, respectively (****, *P* < 0.0001; ***, *P* < 0.001; **, *P* < 0.01; *, *P* < 0.05; ns, not significant). (D) EcN colonization is improved when flies are fed copper. D. melanogaster Canton-S females fed for 24 h on a mixture of sucrose and 1 mM CuCl_2_ or sterile water (as a control), which was applied to a filter disk placed on the fly food surface. Flies were then transferred to new food tubes containing sucrose and either EcN wild-type (WT) or EcN Δ*rclA*. Individuals were collected, surface sterilized, homogenized, and plated on LB agar at various time points to determine bacterial load (CFUs/fly). Statistical analysis was performed with GraphPad Prism using a multiple *t* test with Sidak-Bonferroni correction for multiple comparisons (*n* = 60). Download FIG S5, TIF file, 1.3 MB. Copyright © 2020 Derke et al. 2020 Derke et al. This content is distributed under the terms of the Creative Commons Attribution 4.0 International license.

### RclA protects *E. coli* against the combination of HOCl and intracellular copper.

Next, we sought to investigate how intracellular copper affects the HOCl resistance of *E. coli*. To address this, we grew wild-type and Δ*rclA* mutant E. coli strains overnight in minimal media with or without copper before inoculating the strains into copper-free media to perform HOCl-stress growth curves. Growing overnight cultures in media lacking copper was expected to starve the cells for this metal, thereby reducing the concentration of intracellular copper in those cultures. A broad range of HOCl concentrations were assayed to account for quenching of the oxidant by media components.

**FIG 6:**
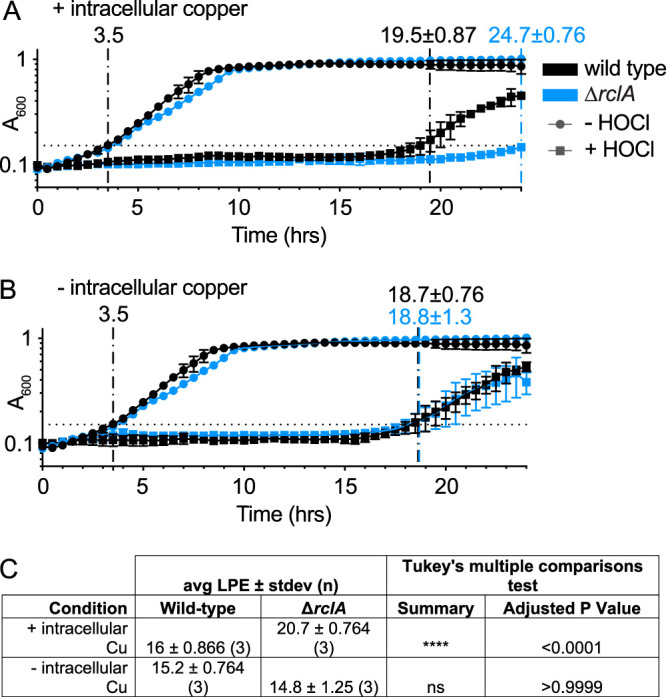
*rclA* is required to resist the combination of HOCl and intracellular Cu. HOCl (132 μM) growth curves (*n* = 3, ± the SD) in copper-free MOPS of wild-type and Δ*rclA* E. coli after being grown overnight in MOPS with (A) or without (B) copper. The time for each sample to reach an average *A*_600_ of 0.15 is indicated by vertical dashed lines. Copper-free MOPS was prepared by treating the media with Chelex-100 chelating resin (Bio-Rad 1421253) and adding back all the metals (metal stock solutions prepared in metal-free water, Optima LC/MS Grade, Fisher Chemical W6-1), except for copper, to the published concentrations (51). In cells containing intracellular copper (A), the Δ*rclA* mutant has delayed growth relative to the wild type; there is no difference between the strains when the cells were starved for intracellular copper (B). One HOCl concentration is shown for simplicity (see Fig. S6 in the supplemental material for growth curves showing more HOCl concentrations and the statistical analysis comparing LPE values between the strains at each condition). (C) Average LPE values for growth curves shown in panels A and B and statistics comparing wild-type and Δ*rclA* strains under all conditions. Differences in average LPE values between the strains were analyzed using two-way ANOVA with Tukey’s multiple-comparison test.

Consistent with the results shown in Fig. 5, E. coli was more sensitive to inhibition by HOCl in media without copper. The Δ*rclA* mutant was more sensitive to HOCl than the wild type when grown overnight in copper-containing media, but this phenotype was lost when cells were starved for copper before stress (Fig. 6). The wild-type strain was also slightly more sensitive to some concentrations of HOCl in the presence of intracellular copper (see Fig. S6B and C), but this difference was much more subtle than in the Δ*rclA* mutant. These results suggest that the physiological role of RclA is to resist the stress resulting from the combination of HOCl and copper in the cytoplasm. They are not consistent, however, with a model where RclA uses Cu to detoxify RCS or other oxidants in the cytoplasm (49), since in that case we would expect the sensitivity of the wild type to decrease to match that of the Δ*rclA* mutant in the absence of Cu, the opposite of what we actually observed (Fig. 6B).

10.1128/mBio.01905-20.8 FIG S6 Comparing LPEs of strains stressed with or without intracellular Cu. (A) Average LPE values for growth curves shown in Fig. 6 and in panels B to E. Differences in average LPE values between the strains were analyzed using a two-way ANOVA with Tukey’s multiple-comparison test. Wild type (B and C) and Δ*rclA* (C and D) E. coli were grown up overnight in MOPS glucose with (D and E) or without copper (A and C). The time for each sample to reach an average *A*_600_ of 0.15 is indicated by vertical dashed lines. HOCl growth curves were performed as described in Fig. 6. Download FIG S6, TIF file, 1.6 MB. Copyright © 2020 Derke et al. 2020 Derke et al. This content is distributed under the terms of the Creative Commons Attribution 4.0 International license.

### RclA reduces copper(II) to copper(I).

Based on the effect of copper starvation on the HOCl sensitivity of the Δ*rclA* mutant, the sequence homology between RclA and MerA, and the predicted oxidoreductase activity of RclA (47), we hypothesized that the substrate of RclA might be copper. The reaction between copper and HOCl is known to generate strong oxidizing intermediates, most likely highly reactive Cu(III) (59–63). HOCl is also capable of oxidizing other transition metals, including iron (64, 65) and manganese (66, 67). We therefore measured the specific activity (SA) of purified RclA in the presence of a panel of biologically relevant metals. We also included mercury in the panel of metals because of RclA’s homology to MerA, although it is unlikely to be physiologically relevant since we do not expect E. coli to encounter this metal in its environment under normal conditions. We note that the oxidized forms of many transition metals are insoluble in aqueous solution, which limited the set of substrates we could test with this experiment.

**FIG 7:**
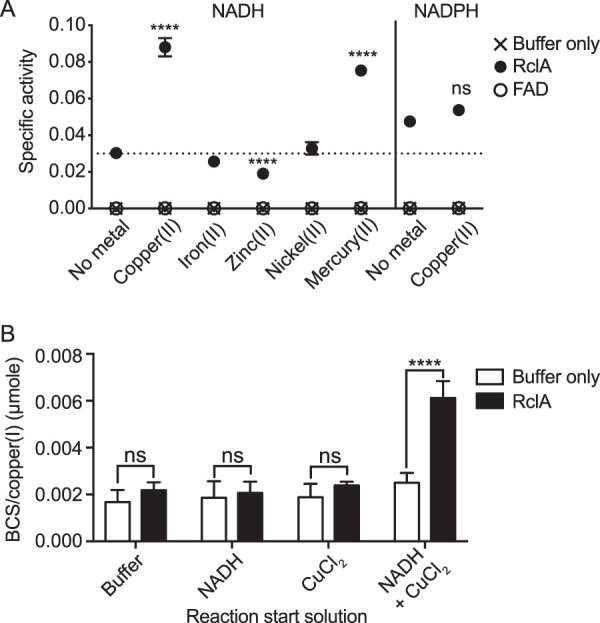
RclA reduces Cu(II) to Cu(I). (A) RclA specific activity (SA) increases in the presence of Cu(II) and Hg(II). SA [μmol of NAD(P)^+^ min^−1^ mg^−1^ RclA] of RclA was assayed by measuring NAD(P)H oxidation over time spectrophotometrically (*n* = 6 for each reaction type, ± the SD). Reactions were started by adding 100 μl of NAD(P)H or NADH and each indicated metal (both to 200 μM final) to 100 μl of RclA (3 μM final) at 37°C using the injector system of a Tecan Infinite M1000 plate reader. All reactions were carried out in 20 mM HEPES–100 mM NaCl (pH 7). NADH absorbance at 340 nm was measured each minute for 5 min. “Buffer only” denotes NAD(P)H oxidation in the presence of the indicated metals, and the “FAD” reactions were performed with 3 μM FAD to control for any possible free cofactor that may contribute to metal reduction in the RclA positive reactions. Differences in SA in the presence of each metal were analyzed using a two-way ANOVA with Dunnett’s multiple-comparison test using the no metal reaction as the control (****, *P* < 0.0001; **, *P* < 0.01). (B) Cu(I) accumulates after the RclA and NADH/copper (II) reaction, as measured by BCS/Cu(I)-complex absorption. Each reaction described in panel A was then stopped at 5 min with 10 μl of a BCS (400 μM final) and EDTA (1 mM final) solution using the injector system of the plate reader. The stopped reaction mixtures were incubated at 37°C for 5 min, with the absorbance of BCS/Cu(I) complex being measured at 483 nm each minute to ensure saturation of BCS. Differences in the amount of BCS/Cu(I) complex between the buffer-only and RclA reactions were analyzed using a two-way ANOVA with Dunnett’s multiple-comparison test using the buffer-only sample as the control for each reaction start solution (****, *P* < 0.0001; ns, not significant).

In the absence of any metal, RclA slowly oxidized NADH (0.0303 μmole NAD^+^ min^−1^ mg^−1^ RclA), consistent with the background activity of other flavin-dependent oxidoreductases in the absence of their specific substrates (68, 69). Three of the metals we tested significantly affected RclA SA, as measured by NADH oxidation. Copper and mercury both significantly increased the SA of RclA, whereas zinc caused a decrease in SA (Fig. 7A). As mentioned above, while the present manuscript was in revision, Baek et al. (49) reported crystal structures of E. coli RclA in the presence or absence of bound copper, along with testing a similar panel of metals as the substrates. The results from that study are consistent with ours, supporting our identification of RclA as Cu(II) reductase. Copper is a potent inhibitor of MerA activity (70), further emphasizing the distinct nature of these two enzymes. RclA oxidized NADPH at similar rates to NADH in the absence of metals, but there was no significant increase to SA when copper was added to the reactions. The addition of exogenous thiols, commonly added as β-mercaptoethanol (BME), is required for MerA activity (71). To determine whether exogenous thiols increase the reaction rate of RclA, we added 1 mM BME to the RclA reactions. BME rapidly reduced Cu(II) to Cu(I) in the absence of RclA but had no effect on the SA of NADH reduction by RclA with or without the addition of copper (see Fig. S7A and B).

10.1128/mBio.01905-20.9 FIG S7 Control experiments for RclA activity. (A) Adding BME to the start solutions of RclA reactions does not increase SA and causes Cu(I) accumulation. (A) RclA reactions performed as in Fig. 7 but with the addition of 1 mM BME for indicated samples (*n* = 6, ± the SD). SA (A) and BCS/Cu(I) (B) concentration determined as in Fig. 7. Differences in SA in the presence of each metal were analyzed using a two-way ANOVA with Dunnett’s multiple-comparison test using the buffer-only sample as the control for each start solution (****, *P* < 0.0001). (C and D) Changes in NADH and copper (I) concentrations over time during the RclA reaction. NADH oxidation (C) and BCS/Cu(I) complex formation (D) were measured over time spectrophotometrically with and without RclA. Reactions were started by adding 100 μl of NADH (200 μM final) or NADH and CuCl_2_ (both 200 μM final) to 100 μl of RclA (3 μM final) at 37°C using the injector system of a Tecan Infinite M1000 plate reader. All reactions were carried out in 20 mM HEPES–100 mM NaCl (pH 7). NADH absorbance at 340 nm was measured each minute for the indicated reaction times. Each reaction was then stopped at the indicated times with 10 μl of a BCS (400 μM final) and EDTA (1 mM final) solution using the injector system of the plate reader (*n* = 6 for each reaction type, ± the SD). The stopped reaction mixtures were incubated at 37°C for 5 min, with the absorbance of BCS/Cu(I) complex being measured at 483 nm each minute to ensure saturation of BCS. Reported BCS/Cu(I) values were calculated using the average of the total absorbance measurements over the 5-min incubation because the values were stable over that time. Differences in the amount of NADH or BCS/Cu(I) complex between the buffer-only and RclA reactions were analyzed using two-way ANOVA with Sidak’s multiple-comparison test (****, *P* < 0.0001; ***, *P* < 0.001; *, *P* < 0.05; ns, not significant). (E) The copper reductase activity of RclA is maintained under anaerobic conditions. BCS/Cu(I) complex concentrations were determined as in Fig. 7, but all reactions were performed in an anaerobic chamber (*n* = 6, ± the SD). Differences in the amount of BCS/Cu(I) complex between the buffer-only and RclA (3 μM) reactions were analyzed using a two-way ANOVA with Tukey’s multiple-comparison test (****, *P* < 0.0001; ns, not significant). (F) The melting temperature (*T_m_*) of RclA is ∼10°C higher than the average *T_m_* of the E. coli proteome. The CD spectra of RclA were measured at the indicated temperatures; 222 nm is indicated by a vertical dashed line. Download FIG S7, TIF file, 1.6 MB. Copyright © 2020 Derke et al. 2020 Derke et al. This content is distributed under the terms of the Creative Commons Attribution 4.0 International license.

Since RclA is an NADH oxidase, the results shown in Fig. 7A strongly suggested that this enzyme was concurrently reducing copper. Copper exists in four possible oxidation states, Cu(I), Cu(II), and the less common and highly reactive Cu(III) and Cu(IV) states (72). The copper salt used in our RclA SA determinations was CuCl_2_, suggesting that RclA was reducing this Cu(II) species to Cu(I). This was initially surprising to us, since Cu(I) is often thought of as a toxic species that causes oxidative stress (54, 73). We therefore first sought to validate that RclA was in fact reducing Cu(II) to Cu(I) while oxidizing NADH to NAD^+^. We measured Cu(I) accumulation in RclA reactions directly using the Cu(I)-specific chelator bathocuproinedisulfonic acid (BCS) (74). NADH spontaneously reduces Cu(II) (75) at rates too slow to impact the measurements made here, but BCS increases the rate of this nonenzymatic copper reduction by shifting the equilibrium of the reaction toward Cu(I) (76). Stopping RclA reactions with a mixture of BCS and EDTA, to chelate any remaining Cu(II), allowed us to observe RclA-dependent Cu(I) accumulation (see Fig. S7C and D). We observed a significant increase in BCS/Cu(I) complex formation only in reaction mixtures containing RclA, NADH, and Cu(II) and not in reaction mixtures lacking any single component (Fig. 7B; see also Fig. S7C and D in the supplemental material). Furthermore, we validated that the copper reductase activity of RclA was maintained when the reactions were performed in an anaerobic chamber (see Fig. S7E) and that FAD alone did not catalyze Cu(II) reduction (see Fig. S7A and B). Taken together, our results show that RclA has Cu(II) reductase activity and directly demonstrate that RclA generates Cu(I) as a product. This is consistent with the findings of Baek et al. (49), who also used site-directed mutagenesis to clarify the roles of the active site cysteines of RclA in Cu(II) reductase activity and binding sensitivity.

### RclA is thermostable and resistant to denaturation by HOCl and urea.

**FIG 8:**
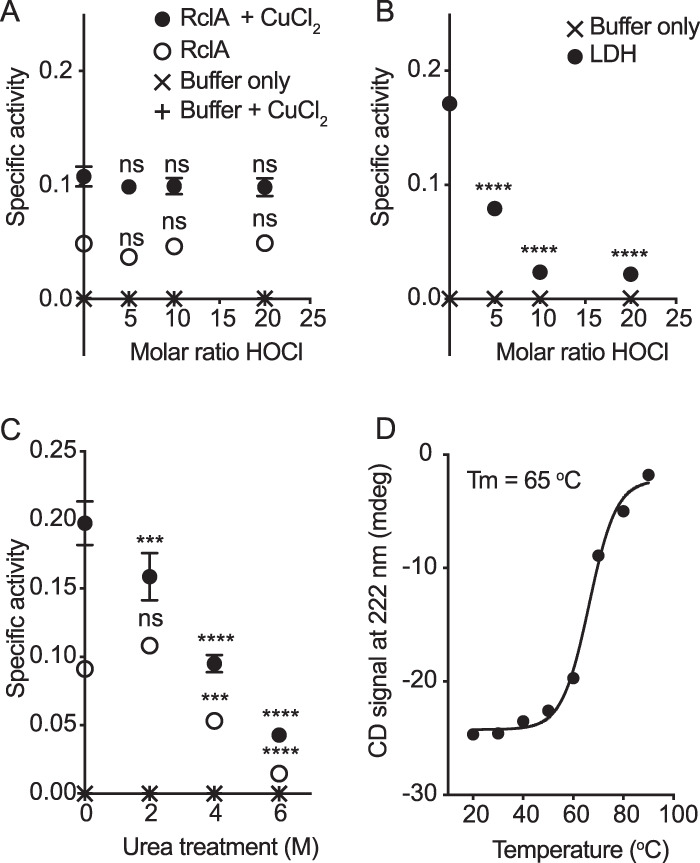
RclA is resistant to denaturation. (A) SA of RclA (μmol NAD^+^ min^−1^ mg RclA^−1^), with or without CuCl_2_, after being treated with the indicated molar ratios of HOCl to RclA. (B) SA of lactate dehydrogenase (LDH) (μmol of NAD^+^ min^−1^ U LDH^−1^) used as control reactions for HOCl degradation of enzymatic activity. (C) RclA with or without CuCl_2_ (200 μM final) after being treated with the indicated concentrations of urea. (D) CD signals at 222 nm (mdeg) (raw data are shown in Fig. S7F in the supplemental material) at each temperature used to determine the *T_m_* of RclA (65°C). Differences in SA (*n* = 6, ± the SD) after treatment were analyzed using two-way ANOVA with Sidak’s multiple-comparison test for HOCl treatment (A and B) and Dunnett’s test using the buffer-only reaction as the control for the urea-treated samples (C) (****, *P* < 0.0001; *, *P* < 0.05; ns, not significant).

We hypothesized that the copper reductase activity of RclA was likely to be relatively stable under denaturing conditions because it must remain active during exposure to HOCl stress, which is known to cause extensive protein misfolding and aggregation *in vivo* (19, 27, 28, 32–34). To test this hypothesis, we first measured RclA activity after treatment with protein denaturing agents (HOCl and urea) *in vitro*. HOCl treatment (with 0-, 5-, 10-, and 20-fold molar ratios of HOCl to RclA) was performed on ice for 30 min, and urea treatment (0, 2, 4, and 6 M) was carried out at room temperature for 24 h. RclA retained full copper reductase activity at all HOCl levels tested, indicating that it is highly resistant to treatment with HOCl (Fig. 8A). By comparison, the NADH oxidase activity of lactate dehydrogenase was significantly decreased after treatment with a 5-fold excess of HOCl (Fig. 8B). RclA also retained a remarkable 35.8% of full activity after being equilibrated in 6 M urea (Fig. 8C). Finally, we used circular dichroism (CD) spectroscopy to measure the melting temperature (*T_m_*) of RclA, which was 65°C (Fig. 8D; see also Fig. S7F), indicating that RclA is thermostable relative to the rest of the E. coli proteome, which has an average *T_m_* of 55°C (standard deviation [SD] = 5.4°C) (60, 77).

### RclA influences copper homeostasis, but HOCl stress does not lead to copper export in wild-type *E. coli*.

Since the copper exporters of E. coli (*copA* and *cusCFBA*) are upregulated by HOCl treatment (Fig. 4; see also Data Set S2 in the supplemental material) (25) and only transport Cu(I) (54, 55, 78, 79), one possible model for how RclA protects against HOCl is that RclA might facilitate the rapid export of cytoplasmic copper, allowing it to react with and eliminate HOCl outside the cell. To test whether the Cu(II) reductase activity of RclA is important for exporting copper during HOCl stress, we measured intracellular copper concentrations in E. coli MG1655 before and after HOCl stress with ICP mass spectrometry (see Fig. S8A). The copper content of the wild type did not change upon HOCl stress, indicating that copper export is not dramatically upregulated under these conditions. We did find that the Δ*rclA* mutant contained, on average, more intracellular copper before HOCl stress than did the wild type but that both strains contained similar amounts of copper after HOCl stress. This suggested that RclA has a role in copper homeostasis under nonstress conditions but that any copper export stimulated by HOCl was RclA independent and, in fact, only occurred in the absence of RclA. To attempt to further probe the effect of intracellular copper on HOCl survival, we constructed mutants lacking *copA*, which is reported to result in increased intracellular copper (55), but unexpectedly found that the *copA rclA* double mutant had a substantial growth defect in copper-free media, even in the absence of HOCl (see Fig. S8B and C). We do not yet know the explanation for this intriguing result, since there are no known essential copper-containing proteins in E. coli (80), although perhaps the most likely candidate under our growth conditions is the Cu-containing cytochrome *bo*_3_ ubiquinol oxidase CyoB, which is involved in aerobic respiration at high O_2_ concentrations (81, 82). Our results suggest that there is considerable complexity in the interactions between Cu homeostasis and RclA under different growth conditions, and future work in our laboratory is focused on exploring these interactions in more detail. However, our current results clearly indicate that Cu is important to understanding bacterial HOCl sensitivity and that the Cu(II) reductase RclA is involved in modulating that process.

10.1128/mBio.01905-20.10 FIG S8 Measuring intracellular copper concentrations during HOCl stress and growth defect of a *rclA copA* double mutant. (A) Intracellular copper concentrations do not change after HOCl stress in wild-type E. coli. E. coli MG1655 wild-type (wt) and Δ*rclA* were grown overnight in MOPS. The next day, both strains were subcultured into MOPS, grown at 37°C with shaking to *A*_600_ = 0.6, and stressed with 400 μM HOCl for 30 min at 37°C with shaking. Intracellular copper before and after HOCl stress of the strains was determined using ICP-MS. Differences between intracellular copper analyzed using two-way ANOVA with Tukey’s multiple-comparison test (*, *P* < 0.05; ns, not significant). (B) A double mutant of *rclA* and *copA* has a growth defect when grown with (B) or without copper (C). HOCl growth curves on cells with or without intracellular copper were performed as described in Fig. 6 with copper-free MOPS glucose (*n* = 3). Download FIG S8, TIF file, 0.7 MB. Copyright © 2020 Derke et al. 2020 Derke et al. This content is distributed under the terms of the Creative Commons Attribution 4.0 International license.

## DISCUSSION

The antimicrobial function of copper in host-microbe interactions is well established (15, 17, 52, 83), although the exact mechanism(s) by which copper kills bacteria remain incompletely known (84, 85). In the present study, we identified a new way in which copper toxicity contributes to host-bacterium interactions via its reactions with RCS. We identified RclA as a highly stable Cu(II) reductase (Fig. 7). This is consistent with the simultaneous report by Baek et al. (49), who also found that both Cu(II) and Hg(II) increase the rate of NADH oxidation by RclA. Importantly, we have now shown that RclA is required *in vivo* for resisting killing by the combination of HOCl and intracellular copper in E. coli (Fig. 6). In the absence of *rclA*, E. coli had a significant defect in initial colonization that was partially eliminated when production of HOCl by the host was reduced (Fig. 2). The amount of copper in bacterial cells is low (15, 16), but how much is unbound by protein and its redox state under different conditions are unknown (15). Given the broad conservation of RclA among host-associated microbes, we propose that there is likely to be a common and previously unsuspected role for copper redox reactions in interactions between bacteria and the innate immune system.

Copper accumulates in host tissues during inflammation (86, 87), as do RCS (88, 89). Our discovery that even very low concentrations of extracellular copper can protect bacteria against RCS both *in vitro* and *in vivo* adds a new and important facet to understanding copper’s role in innate immunity. Since a large proportion of host tissue damage during inflammation is due to HOCl (90, 91), the presence of copper in inflamed tissues may play an important role not only in killing bacteria but potentially also in protecting host cells, although this hypothesis will require further testing. Our results also show that media copper concentrations are a key variable in experiments testing the sensitivity of cells to HOCl and that care must be taken to account for media copper content and use metal-free culture vessels in such experiments.

Both HOCl and copper can cause oxidative stress in bacteria and Cu(I) is generally considered more toxic than Cu(II) (15, 19, 52, 83, 84, 92), so we were initially surprised that a Cu(II) reductase protected E. coli against HOCl. Copper reacts with the ROS hydrogen peroxide (H_2_O_2_) to form highly reactive hydroxyl radicals *in vitro* (17, 53, 73, 85), but there is also strong evidence that oxidation is not the major cause of Cu toxicity in E. coli (85, 93). CusRS and CueR are exceptionally sensitive to changes in Cu concentrations in the periplasm and cytoplasm, respectively (94, 95). CueR, for example, has zeptomolar Cu binding affinity (95). The upregulation of the CusRS and CueR regulons under HOCl stress (Fig. 4) indicates that free Cu is increasing in both the cytoplasm and the periplasm, which could plausibly result from the oxidation of cysteine and histidine residues in Cu-binding proteins by HOCl (96, 97). Redox proteomics of RCS-stressed E. coli (98, 99) have not identified oxidized Cu-binding proteins, but the methods used in those studies to date are limited to detection of the most common proteins in the cell. Detection of oxidation in less abundant proteins will require more specialized methods (30).

**FIG 9:**
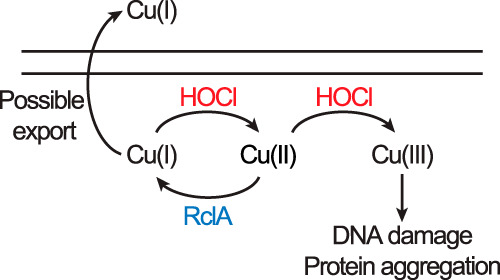
Proposed model for RclA activity in reducing the toxicity of HOCl. Oxidation of Cu by HOCl can result in the production of the highly-reactive and unstable Cu(III) (72, 110). Limiting the amount of cytoplasmic Cu(II) available would prevent the accumulation of Cu(III) during HOCl stress, thereby reducing the toxicity. Converting intracellular Cu(II) to Cu(I) may also facilitate Cu export via CopA(Z) mediated export systems (55).

How the presence of copper influences bacterial sensitivity to RCS has not been investigated before this study, but the chemistry of reactions between HOCl and copper is complicated and different from that of reactions between ROS and Cu. HOCl can oxidize Cu(II) to highly reactive Cu(III) (59–63), and both Cu(I) and Cu(II) are known to catalyze the breakdown of HOCl (59–62). At nearly neutral pH, similar to that in the large intestine or bacterial cytoplasm, Cu(I) accelerates the rate of decomposition of HOCl to O_2_ and chloride ions by as much as 10^8^-fold (60). One possibility to explain the protective effect of RclA is that it might facilitate an HOCl-degrading Cu(I)/Cu(II) redox cycle in the cytoplasm. Alternatively, Baek et al. (49) note the increased O_2_ consumption by RclA in the presence of Cu(II), and propose that RclA protects against oxidative stress by lowering O_2_ levels. If either of these were the case, however, RclA would require copper to drive HOCl resistance and the Δ*rclA* mutant would become more sensitive to HOCl in the absence of copper, the opposite of what we actually observed (Fig. 6). We therefore propose that RclA-catalyzed reduction of Cu(II) to Cu(I) may act to limit the production of Cu(III) in the cytoplasm (Fig. 9). Uncontrolled production of Cu(III) could greatly potentiate the ability of HOCl to kill bacterial cells Alternatively, RclA may also ensure that intracellular copper remains in the Cu(I) state, where it can be bound by Cu chaperones like CopA(Z) (100). All of the known proteins involved in Cu homeostasis and export in the E. coli cytoplasm are specific to Cu(I) (52, 54, 55). Although our data (see Fig. S8A in the supplemental material) indicate that Cu export is not detectably upregulated in HOCl-stressed wild-type cells, we cannot rule out a role for RclA in maintaining Cu homeostasis under nonstress conditions, especially given the unexpected phenotype of a *copA rclA* double mutant (see Fig. S8B and C). This is an active area of research in our lab, and we are currently exploring how RclA and the various Cu homeostasis mechanisms of E. coli interact both under nonstress conditions and in the presence of HOCl.

The rate at which RclA oxidized NADH in the presence of copper *in vitro* was slow (approximately 4.4 min^−1^) (Fig. 7) (49), suggesting that we have not yet identified optimal reaction conditions for this enzyme. However, expression of *rclA* is rapidly induced >100-fold after sublethal doses of HOCl in E. coli (Fig. 4; see Data Set S2 in the supplemental material) (26), which could compensate *in vivo* for the low rate of NADH turnover we observed *in vitro*.

While RclA itself is widely conserved, the *rclABCR* locus as a whole is restricted to certain enteric proteobacteria, including E. coli, *Salmonella*, *Citrobacter*, *Raoultella*, *Serratia*, and *Shigella*. These genera are notable for their close association with gut inflammation and the ability of pathogenic strains to bloom to very high levels in the gut in disease states (2, 3, 7–10, 101, 102). We hypothesize that the ability to survive increased levels of antimicrobial compounds (including RCS) in the inflamed gut is important for the ability of enterobacteria to exploit this niche, and our *in vivo* results with the Δ*rclA* mutant generally support this idea (Fig. 2). Many noninflammatory commensal bacteria do encode *rclA* homologs (Fig. 1; see Data Set S1 in the supplemental material), including members of the *Bacteroidetes*, *Clostridiaceae*, and *Lactobacillaceae*, where their physiological roles are unknown. Expression of the *rclA* homolog of the probiotic Lactobacillus reuteri is induced modestly by HOCl, but an L. reuteri *rclA* mutant is not sensitive to HOCl stress (36). It is unclear, however, whether this is because RclA has a different physiological function in L. reuteri (possibly related to Cu homeostasis) or because RclA requires either strong induction or the presence of RclB and RclC to protect against HOCl under laboratory growth conditions. We do not currently know the physiological roles of RclB, which is a small predicted periplasmic protein, or RclC, which is a predicted inner membrane protein, although deletion of either of these genes results in increased HOCl sensitivity in E. coli (26). We hypothesize that they may form a complex with RclA *in vivo* and enhance its copper-dependent protective activity and are currently pursuing experiments to test this idea.

## MATERIALS AND METHODS

### Strain and plasmid construction.

E. coli strain MJG0586 [F^–^ λ^–^ *rph-1* Δ*ilvG rfb-50* Δ*rclA* λ(DE3 [*lacI lacUV5*-T7 gene 1 *ind1 sam7 nin5*)] was generated from MJG0046 (F^–^ λ^–^ *rph-1* Δ*ilvG rfb-50* Δ*rclA*) (26) using the Novagen DE3 lysogenization kit, according to the manufacturer’s instructions. The Δ*copA767*::*kan*^+^ allele from the Keio collection (103) was transduced into E. coli strains MG1655 and MJG0046 by P1 transduction (104), yielding strains MJG1759 (F^–^ λ^–^ *rph-1* Δ*ilvG rfb-50* Δ*copA767*::*kan*^+^) and MJG1760 (F^–^ λ^–^ *rph-1* Δ*ilvG rfb-50* Δ*rclA* Δ*copA767*::*kan*^+^) The RclA coding sequence (1374 bp) was amplified from E. coli MG1655 genomic DNA with the primers 5′ CTC GGT CTC CAA TGA ATA AAT ATC AGG CAG TGA 3′ and 5′ CTC GGT CTC AGC GCT TTA TTT GAC TAA TGA AAA TAG ATC A 3′ and cloned into the Eco31I (BsaI) sites of plasmid pPR-IBA101 (IBA Life Sciences) to yield plasmid pRCLA10. pRCLA11 was generated by using QuikChange site directed mutagenesis (Agilent), modified to use a single mutagenic primer (5′ CTA TTT TCA TTA GTC AAA AGC GCT TGG AGC CAC CC 3′), to remove the stop codon between the RclA coding sequence and the twin-strep tag sequence in pRCLA10. E. coli Nissle 1917 Δ*rclA*::*cat^+^* strain MJG0846 was made by recombineering (105) using primers 5′ CGT CTA TAG TCA TGA TGT CAA ATG AAC GCG TTT CGA CAG GAA ATC ATC ATG GTG TAG GCT GGA GCT GCT TC 3′ and 5′ CTT TTC TCT GAG ACG CCA GAA TAT TTG TTC TGG CGT CTG ATT TTG AGT TTA CAT ATG AAT ATC CTC CTT AG 3′ to amplify the chloramphenicol resistance cassette from pKD3. The *cat*^+^ insertion was resolved using pCP20 resulting in the E. coli Nissle 1917 Δ*rclA* strain MJG0860.

### Protein expression and purification.

Expression of twin-strep-tagged RclA was done in MJG0586 containing pRCLA11 (MJG1338) in M9 minimal media (106) containing 2 g liter^−1^ glucose and 100 μg ml^−1^ ampicillin. Overnight cultures of MJG1338 were diluted 1:100 and grown to an *A*_600_ of 0.4 at 37°C with shaking. When the *A*_600_ reached 0.4, expression was induced with IPTG (isopropyl-β-d-thiogalactopyranoside; 1 mM final concentration) and allowed to continue for 12 to 18 h at 20°C. Purification of recombinant RclA was achieved to high purity using a 1-ml StrepTrap HP column (GE, 28-9136-30 AC) according to the manufacturer’s instructions. Purified protein was subsequently saturated with FAD cofactor by incubating the protein preparation with a 10-fold molar excess of FAD at room temperature for 45 min. Excess FAD and elution buffer were dialyzed away with three exchanges of 1 liter of RclA storage buffer (50 mM Tris-HCl [pH 7.5], 0.5 M NaCl, 2 mM dithiothreitol, 10% glycerol) at 4°C overnight.

### Colonization of *D. melanogaster* with *E. coli* Nissle 1917.

**(i) *D. melanogaster* stocks and husbandry.** Canton-S flies were used as a wild-type line. Duox-RNAi flies were obtained from crosses of *UAS-dDuox-RNAi* with *NP1-GAL4* (gut-specific driver). Unless otherwise noted, the flies were reared on cornmeal medium (per liter of medium: 50 g yeast, 70 g cornmeal, 6 g agar, 40 g sucrose, 1.25 ml methyl-Paraben, and 5 ml 95% ethanol).

**(ii) Oral infections.** Adult female flies were starved for 2 h at 29°C prior to being fed a 1:1 suspension of bacteria (optical density at 600 nm [OD_600_] = 200) and 2.5% sucrose applied to a filter paper disk on the surface of fly food. Suspensions of bacteria at an OD_600_ of 200 were consistently found to contain 1 × 10^11^ CFU/ml. A negative control was prepared by substituting Luria-Bertani (LB) medium for the bacterial suspension. Infections were maintained at 29°C.

**(iii) CFU determination.** Flies were surface sterilized in 70% ethanol and rinsed in sterile phosphate-buffered saline (PBS). Individual flies were homogenized in screw-top bead tubes containing 600 μl of PBS. Dilution series of homogenates were prepared with a range of (10^0^ to 10^−5^). Spots (3 μl) of each dilution were applied to LB plates and grown overnight at 30°C. E. coli colonies were identified by morphology and counted to determine the CFU/fly.

### RNA sequencing.

E. coli Nissle 1917 was grown anaerobically (90% N_2_, 5% CO_2_, and 5% H_2_, maintained with a Coy Laboratory Products anaerobic chamber) at 37°C in MOPS minimal media (Teknova) in sealed Hungate tubes to an *A*_600_ of 0.25, then treated anaerobically with 0.4 mM HOCl. Samples (7 ml) were harvested into 7 ml of ice-cold isopropanol immediately before and 15 or 30 min after HOCl addition, harvested by centrifugation, and stored at –80°C until use. RNA was purified using a RiboPure-Bacteria kit (Ambion), according to the manufacturer’s instructions. mRNA sequencing was performed at the UAB Heflin Center for Genomic Sciences using an Illumina NextSeq 500 as described by the manufacturer (Illumina, Inc.). Use of an Agilent SureSelect strand-specific mRNA library kit (Agilent) and ribosome reduction with the RiboMinus protocol for Gram-negative and Gram-positive bacteria (Life Technologies) were performed as described by the manufacturers. The resulting mRNA was randomly fragmented with cations and heat, followed by first-strand synthesis using random primers. Second-strand cDNA production was done with standard techniques, and cDNA libraries were quantitated using qPCR in a Roche LightCycler 480 with a Kapa Biosystems kit for Illumina library quantitation prior to cluster generation, which was performed according to the manufacturers’ recommendations for onboard clustering (Illumina). We generated approximately 8 million double-stranded 50-bp reads per sample. Data were analyzed using Bowtie2 and DEseq2. RNA sequencing data have been deposited in NCBI’s Gene Expression Omnibus (107) and are accessible through GEO series accession number GSE144068.

### Growth curves for measuring sensitivity to HOCl.

The molar HOCl concentration from concentrated sodium hypochlorite (Sigma-Aldrich, catalog no. 425044) was quantified by measuring the *A*_292_ of the stock solution diluted in 10 mM NaOH. Copper-free MOPS was prepared by removing total metals from MOPS minimal media (Teknova) containing 2 g liter^−1^ glucose and 1.32 mM K_2_HPO_4_ by treating prepared media with a universal chelator (Chelex 100 chelating resin; Bio-Rad catalog no. 1421253), filtering sterilizing away the Chelex resin, and adding back the metals normally present in MOPS suspended in metal-free water, except for copper. Overnight cultures of MG1655 and MJG0046 were grown up overnight in MOPS with or without copper. The overnight cultures were normalized to an *A*_600_ of 0.8 in and diluted to an *A*_600_ of 0.08 in MOPS minimal medium without copper using the indicated combinations and/or concentrations of HOCl and CuCl_2_. Cultures were incubated with shaking at 37°C in a Tecan Infinite M1000 plate reader with the *A*_600_ being measured every 30 min for 27 h. Sensitivity was subsequently determined by comparing lag-phase extensions (the difference in hours to reach *A*_600_ ≥ 0.15 from the no HOCl treatment control under the same CuCl_2_ condition) for each stress condition.

### Determining the effect of copper on lethal HOCl stress.

Wild-type and Δ*rclA* E. coli MG1655 strains were grown overnight in MOPS minimal media. Next, 500 μl of the overnight culture was pelleted, resuspended in copper-free MOPS, and subcultured into 9.5 ml of copper-free MOPS, followed by growth with shaking at 37°C to mid-log phase (*A*_600_ = 0.3 to 0.4). Once mid-log phase was reached, 1 ml of cells was aliquoted into microcentrifuge tubes for each treatment. The cells were treated by adding the indicated amounts of copper (II) chloride, followed by 1 mM HOCl, and then incubated on a 37°C heat block for 10 min. After incubation, treatments were serially diluted in PBS, and 5-μl aliquots were spotted onto LB agar plates. Plates were dried and incubated at room temperature for 2 days.

### Measuring intracellular copper after HOCl stress.

Wild-type and Δ*rclA* E. coli strains were grown to an *A*_600_ of 0.6 in MOPS medium and stressed with 400 μM HOCl for 30 min at 37°C with shaking. After 30 min, the cultures were diluted 2-fold with MOPS medium to quench HOCl and then pelleted. The mass of pellets was determined after three rinses with PBS. The amount of copper was measured via inductively coupled plasma mass spectrometry (7700× ICP-MS; Agilent Technologies, Santa Clara, CA) after suspension of the collected pellets in concentrated nitric acid and dilution of the suspensions to a 2% nitric acid matrix. Samples were filtered through 0.22-μm polytetrafluoroethylene filters to remove any particulates before running metal determinations. Copper concentrations were determined by comparison to a standard curve (Agilent, catalog no. 5188-6525) as calculated by Agilent software (ICP-MS MassHunter v4.3). Values determined by ICP-MS were normalized to pellet mass and dilution factor.

### NADH oxidase activity.

Activity of purified RclA was assayed in 20 mM HEPES–100 mM NaCl (pH 7) by measuring NADH oxidation over time spectrophotometrically. Purified recombinant RclA was preloaded into wells of a 96-well plate (100 μl of 6 μM RclA). Reactions were started by adding 100 μl of NADH (200 μM final) with the indicated metal salts (200 μM final) to RclA (3 μM final) at 37°C. The absorbance of NADH (*A*_340_) was measured kinetically each minute for 5 min in a Tecan Infinite M1000 plate reader. *A*_340_ values were then used to calculate the μmol of NADH (ε_340_ = 6,300 M^−1^ cm^−1^) and NADPH (ε_340_ = 6,220 M^−1^ cm^−1^) at each time point. The absolute values of slopes of NAD(P)H (μmol) over time (min) were divided by the amount (mg) of RclA used to determine the reported specific activities [SA; μmol NAD(P)^+^ min^−1^ mg^−1^ RclA] for each condition.

### Copper(I) quantification.

Cu(I) accumulation after the course of the RclA reaction was measured by using bathocuproinedisulfonic acid disodium salt (BCS; Sigma-Aldrich, B1125). NADH oxidation reactions were carried out as in the previous section but were started with either NADH (200 μM final), CuCl_2_ (200 μM final), NADH and CuCl_2_ (both at 200 μM final), or reaction buffer as a negative control to observe levels of background copper in the reagents used. Each reaction was stopped at 5 min with a BCS (400 μM final) and EDTA (1 mM final) solution using the injector system of a Tecan Infinite M1000 plate reader. The stopped reaction mixtures were incubated at 37°C, with the absorbance of BCS/Cu(I) complex being measured at 483 nm every minute for 5 min to ensure complete saturation of the BCS. The amount of the BCS/Cu(I) complex after 5 min was determined using the molar extinction coefficient 13,000 M^−1^ cm^−1^ (74).

### NADH oxidase activity after treatment with urea and HOCl.

The SA of RclA after being treated with denaturing agents was measured by determining the SA as described above after incubation of RclA with either urea or HOCl. Urea treatment was done on 3 μM RclA for 24 h at room temperature with increasing concentrations of urea (0, 2, 4, and 6 M) in reaction buffer (20 mM HEPES, 100 mM NaCl [pH 7]). HOCl treatment was done by mixing increasing molar ratios of HOCl (0, 5, 10, and 20×) with concentrated RclA or l-lactate dehydrogenase (LDH; Sigma, LLDH-RO 10127230001; 35 μM) in oxidation buffer (50 mM sodium phosphate [pH 6.8], 150 mM NaCl) and incubating the mixtures on ice for 30 min. HOCl treatment was quenched after 30 min by diluting the RclA solutions to 6 μM and the LDH solutions to 1 μM in reaction buffer (20 mM HEPES, 100 mM NaCl [pH 7]). NADH oxidation reactions with LDH were performed under the same conditions as the RclA reactions but were started with 1.2 mM NADH and 1.2 mM pyruvate.

### Melting temperature determination.

CD spectra were obtained on a Jasco J815 circular dichroism spectrometer. CD spectra were collected on purified recombinant RclA exchanged into 20 mM HEPES and 100 mM NaCl (pH 7.5). Room temperature CD spectra in the range of 260 to 190 nm were obtained in 0.1-mm demountable quartz cells. Thermal CD data between 30 and 90°C were obtained in standard 1.0-mm quartz cells. All data were collected with a 1.0-nm step size, an 8-s averaging time per point, and a 2-nm bandwidth. Data were baseline corrected against the appropriate buffer solution and smoothed with Jasco software.

### Data analysis and bioinformatics.

**(i) Statistics.** All statistical analyses were performed using GraphPad Prism (v7.0a).

**(ii) Phylogenetic tree.** An RclA conservation tree was made from amino acid sequence alignments of RclA (BLAST E-value < 1 × 10^−90^ in 284 species), RclB (BLAST E-value < 1 × 10^−1^ in 61 species), RclC, (BLAST E-value < 1 × 10^−80^ in 49 species), and RclR (BLAST E-value < 1 × 10^−40^ in 43 species). BLAST searches were done by comparing to each respective protein in E. coli MG1655. A tree graphic was made using the Interactive Tree of Life (108) (https://itol.embl.de/).

**(iii) Amino acid sequence alignments.** Active site alignment of E. coli RclA (ADC80840.1) and MerA amino acid sequences was done for seven bacterial species (Escherichia coli, ADC80840.1; Staphylococcus aureus, AKA87329.1; Salmonella enterica, ABQ57371.1; Listeria monocytogenes, PDA94520.1; Klebsiella pneumoniae, ABY75610.1; and Serratia marcescens, ADM52740.1). Alignment was done using CLUSTAL O (1.2.4; www.ebi.ac.uk/Tools/msa/clustalo/), and a graphic was prepared using WEBLOGO (weblogo.berkeley.edu/logo.cgi). A full-length alignment of E. coli RclA (ADC80840.1) and MerA (ADC80840.1) was also prepared. Alignment, conservation scoring, and graphic were done using PRALINE (www.ibi.vu.nl/programs/pralinewww/).

### Data accessibility.

All strains generated in the course of this study are available from the authors upon request.
