# Supplementary figures and images for: The Cu(II) Reductase RclA Protects Escherichia coli against the Combination of Hypochlorous Acid and Intracellular Copper
Source: mBio. 2020 Sep 29;11(5):e01905-20. doi: 10.1128/mBio.01905-20 (PMC7527725; doi:10.1128/mBio.01905-20)

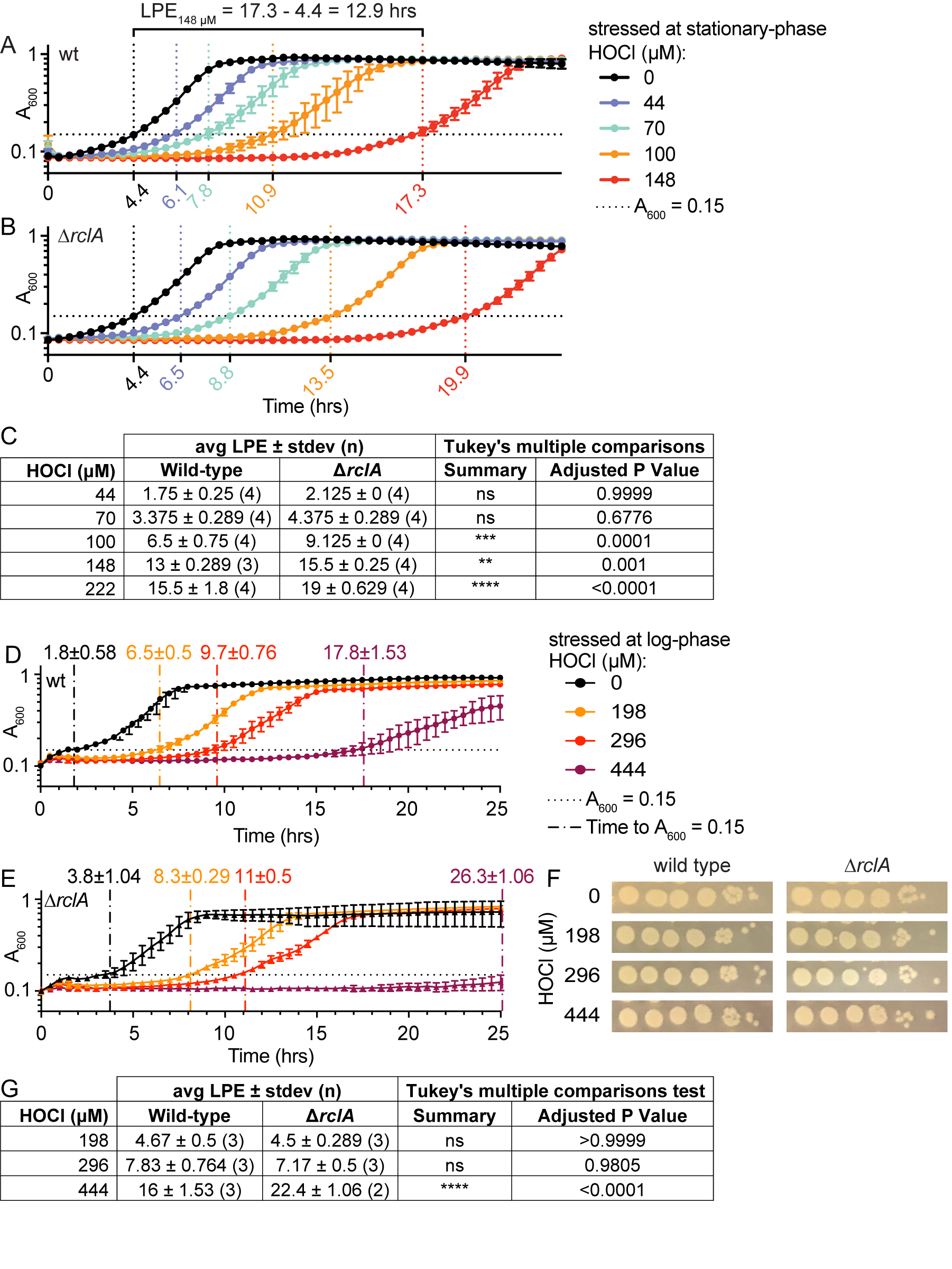

Supplement: FIG S1 [file mBio.01905-20-sf001.tif]

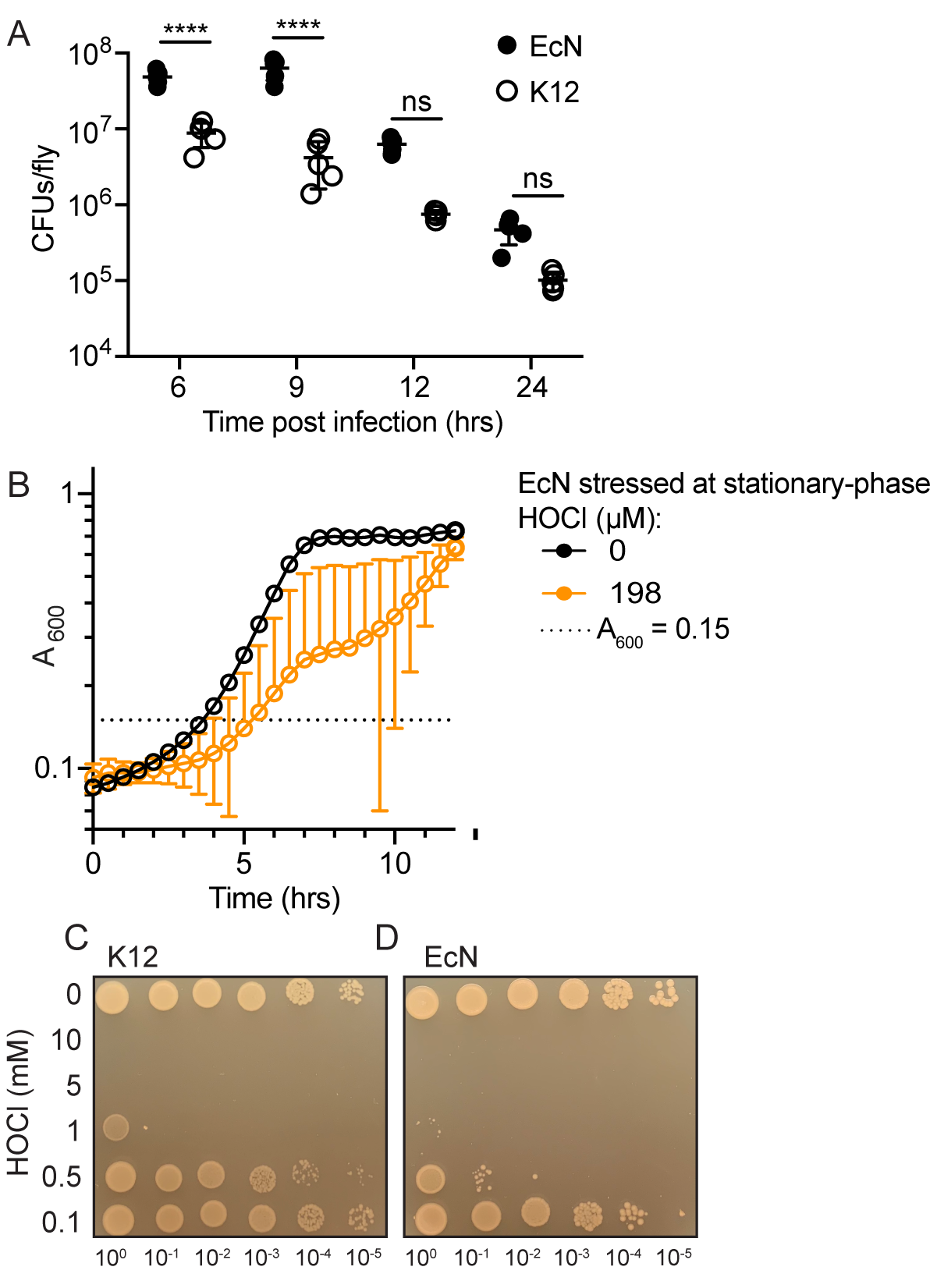

Supplement: FIG S2 [file mBio.01905-20-sf002.tif]

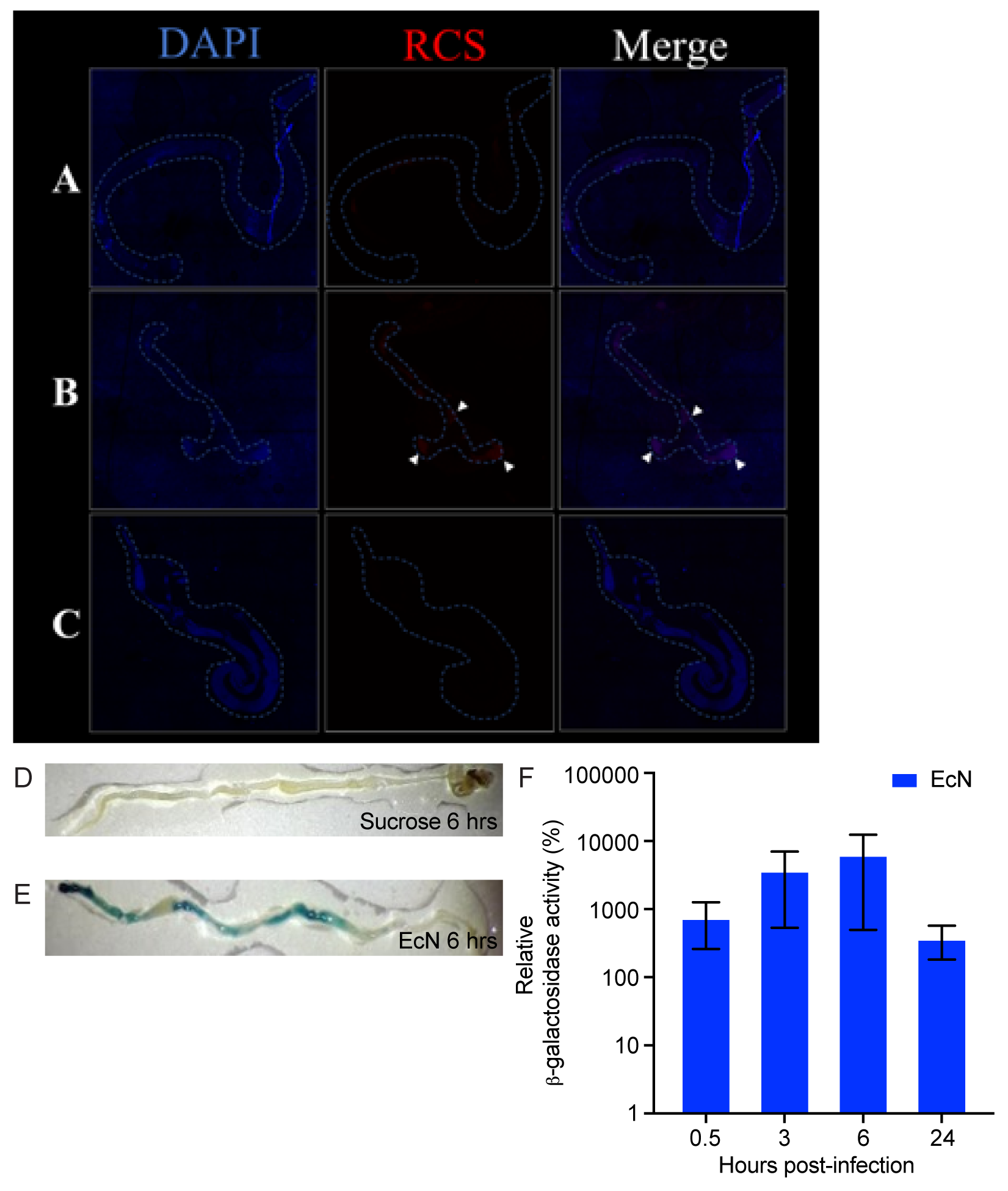

Supplement: FIG S3 [file mBio.01905-20-sf003.tif]

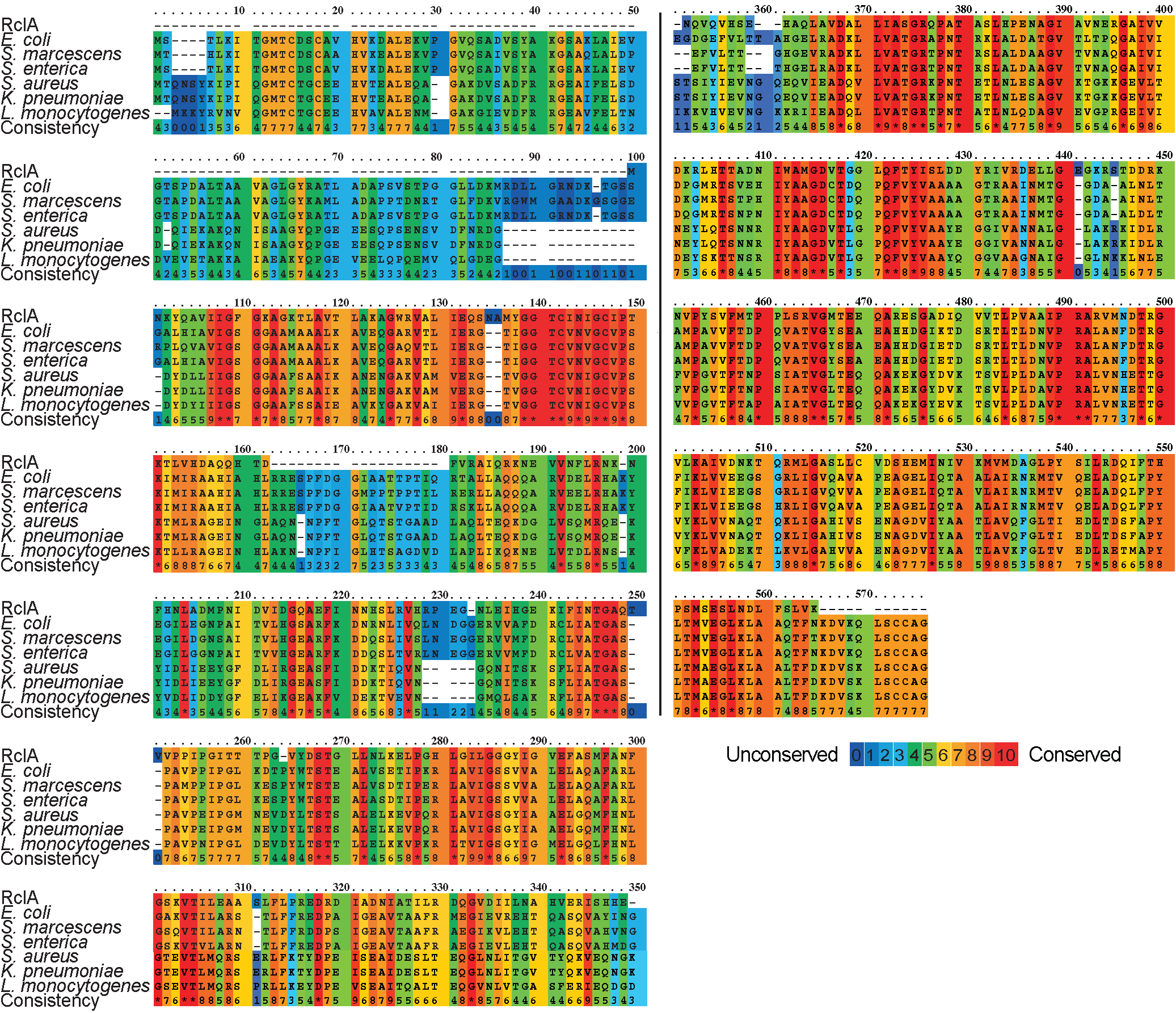

Supplement: FIG S4 [file mBio.01905-20-sf004.tif]

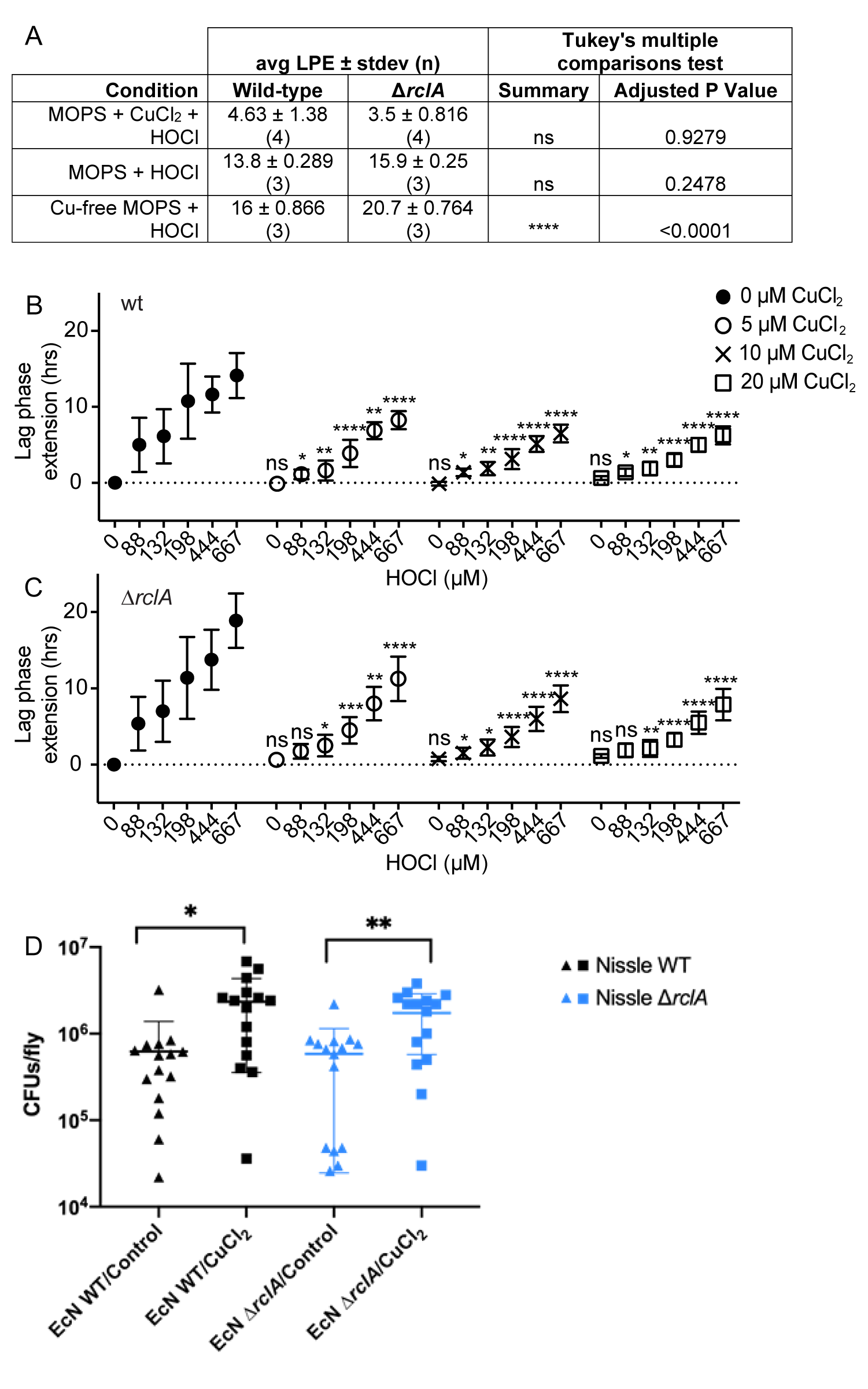

Supplement: FIG S5 [file mBio.01905-20-sf005.tif]

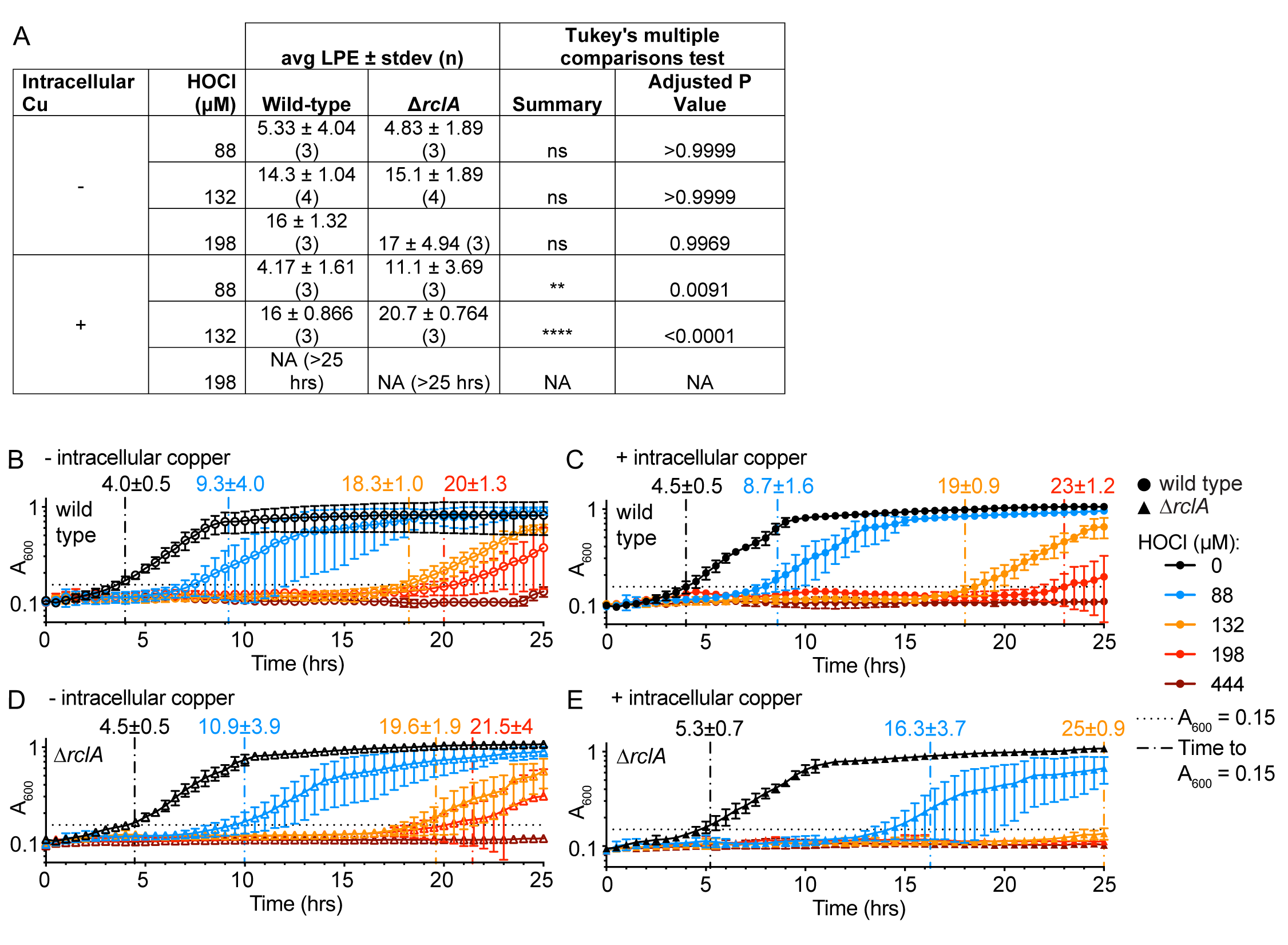

Supplement: FIG S6 [file mBio.01905-20-sf006.tif]

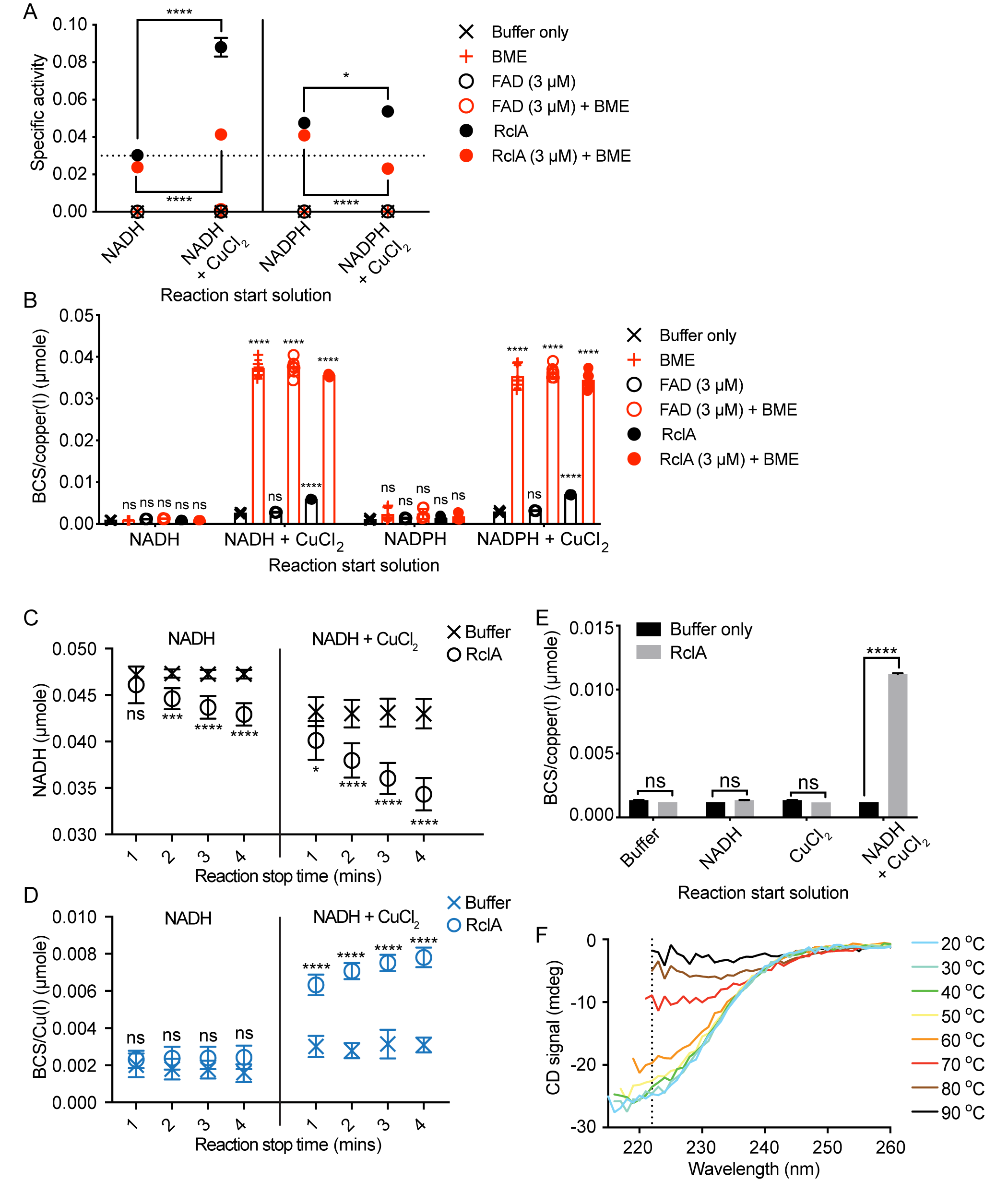

Supplement: FIG S7 [file mBio.01905-20-sf007.tif]

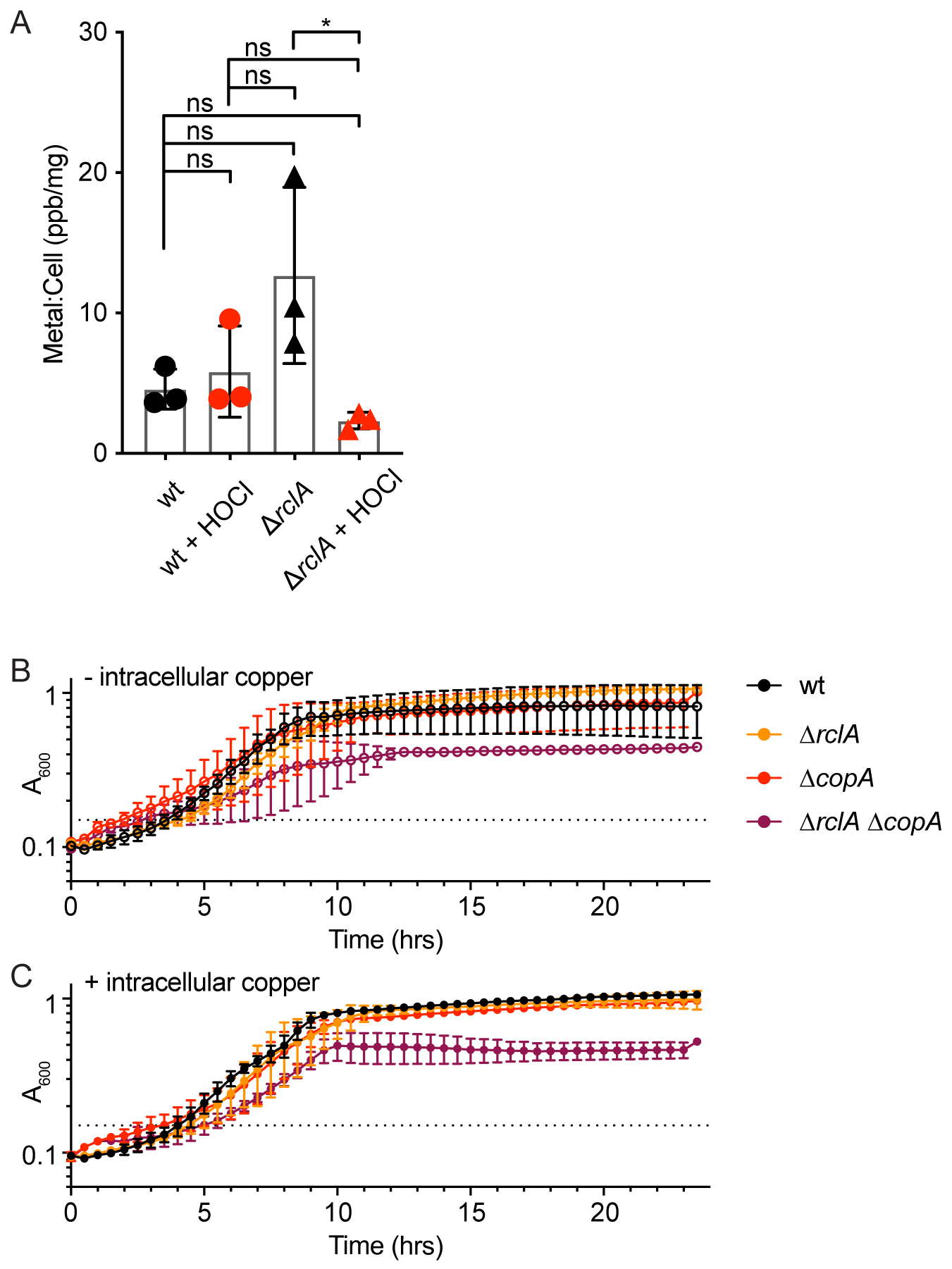

Supplement: FIG S8 [file mBio.01905-20-sf008.tif]
